# Hippo signaling and histone methylation control cardiomyocyte cell cycle re-entry through distinct transcriptional pathways

**DOI:** 10.1371/journal.pone.0281610

**Published:** 2023-02-13

**Authors:** Zhenhe Zhang, Miles Freeman, Yiqiang Zhang, Danny El-Nachef, George Davenport, Allison Williams, W. Robb MacLellan

**Affiliations:** 1 Cardiology Division, Department of Medicine, University of Washington, Seattle, Washington, United States of America; 2 Center for Cardiovascular Biology, University of Washington, Seattle, Washington, United States of America; 3 Institute for Stem Cell and Regenerative Medicine, University of Washington, Seattle, Washington, United States of America; 4 Department of Anatomy, Biochemistry and Physiology, John A. Burn School of Medicine, University of Hawaii, Honolulu, Hawaii, United States of America; Indiana University School of Medicine, UNITED STATES

## Abstract

**Aims:**

Accumulating data demonstrates that new adult cardiomyocytes (CMs) are generated throughout life from pre-existing CMs, although the absolute magnitude of CM self-renewal is very low. Modifying epigenetic histone modifications or activating the Hippo-Yap pathway have been shown to promote adult CM cycling and proliferation. Whether these interventions work through common pathways or act independently is unknown. For the first time we have determined whether lysine demethylase 4D (KDM4D)-mediated CM-specific H3K9 demethylation and Hippo pathways inhibition have additive or redundant roles in promoting CM cell cycle re-entry.

**Methods and results:**

We found that activating Yap1 in cultured neonatal rat ventricular myocytes (NRVM) through overexpressing Hippo pathway inhibitor, miR-199, preferentially increased S-phase CMs, while H3K9me3 demethylase KDM4D preferentially increased G2/M markers in CMs. Together KDM4D and miR-199 further increased total cell number of NRVMs in culture. Inhibition of Hippo signaling via knock-down of Salvador Family WW Domain Containing Protein 1 (Sav1) also led to S-phase reactivation and additional cell cycle re-entry was seen when combined with KDM4D overexpression. Inducible activating KDM4D (iKDM4D) in adult transgenic mice together with shRNA mediated knock-down of Sav1 (iKDM4D+Sav1-sh) resulted in a significant increase in cycling CMs compared to either intervention alone. KDM4D preferentially induced expression of genes regulating late (G2/M) phases of the cell cycle, while miR-199 and si-Sav1 preferentially up-regulated genes involved in G1/S phase. KDM4D upregulated E2F1 and FoxM1 expression, whereas miR-199 and si-Sav1 induced Myc. Using transgenic mice over-expressing KDM4D together with Myc, we demonstrated that KDM4D/Myc significantly increased CM cell cycling but did not affect cardiac function.

**Conclusions:**

KDM4D effects on CM cell cycle activity are additive with the Hippo-Yap1 pathway and appear to preferentially regulate different cell cycle regulators. This may have important implications for strategies that target cardiac regeneration in treating heart disease

## Introduction

In mammals, cardiomyocytes (CMs) proliferate rapidly during embryonic development then exit the cell cycle shortly after birth [1]. However, increasing evidence demonstrates that pre-existing CMs are the source of CM renewal through proliferation in adult hearts but only do so at a very low rate throughout life [1–3]. Therefore, enhancing adult CM (ACM) proliferation is an attractive strategy to compensate for the lost CMs in injured myocardium [4, 5]. Numerous factors have been analyzed for their effect on CM proliferation. Fibroblast growth factor 1 (FGF1)/p38 MAP kinase inhibitor in post-infarct rats increased CM proliferation, reduced scarring, and markedly improved cardiac function [6]. Oncostatin M (OSM) and IL-13 stimulate ACM cell cycle re-entry and improve cardiac function after myocardial infarction (MI) through Raf/MEK/Erk and STAT3/6 signaling pathway [7, 8]. Extracellular matrix (ECM) components such as periostin and agrin have also been reported to regulate CM proliferation and may provide a therapeutic target for advanced heart failure [9, 10]. The utility of these approaches remain uncertain but the concept of promoting endogenous myocardial proliferation and regeneration is a promising strategy to prevent the development of heart failure after myocardial injury.

The mechanisms through which these pro-growth factors exert their effects on CMs is poorly understood but several miRNAs have also been reported to promote CMs proliferation [11–14]. A high-throughput screen of 875 miRNAs found at least 40 miRNAs that increased both DNA synthesis and cytokinesis in cultured neonatal mouse and rat CMs [15]. In particular, miR-590 and miR-199a could promote cell-cycle re-entry and stimulate CM proliferation in both neonatal and adult rat CMs. Loss of miR-302-367 led to decreased CM proliferation during development while miR-302-367 overexpression resulted in a marked increase in CM cycling, in part through the repression of the Hippo signaling pathway [16]. The majority of miRNAs that impact CM cycling appear to exert their effect through the Hippo signaling pathway [16–18]. The Hippo pathway signaling cascade plays an essential role in organ size control from Drosophila to mammals by regulating cell proliferation, apoptosis, and stem cell/progenitor cell fate determination [19–21]. And the core components of the Hippo pathway are highly conserved in mammals, including the Mst kinases (MST1/2), the adaptor Salvador (Sav1), and phosphorylate Lats kinase (LAT1/2) [22–24]. Binding to Sav1 activates MST1/2 which phosphorylates LATS1/2. The active LATS1/2 phosphorylates and inhibits the downstream transcription cofactors Yap. When inhibiting Hippo pathway, Yap enters the nucleus, where it cooperates with TEAD family transcription factors to activate target genes [25–27].

Cell cycle gene transcription is also regulated via complex epigenetic signaling. In particular, methylation of histone H3 can activate or repress transcription [28, 29]. For example, H3K4me1 and H3K4me3 mark activate chromatin, whereas H3K9me3 and H3K27me3 mark silence chromatin [30, 31]. These histone modifications are tightly controlled by histone methyltransferases (HMTs), and histone demethylases (HDMs) enzymes [32–34]. Recently, our lab demonstrated that depletion of H3K9me3 by KDM4D in ACMs results in remarkable transcriptomic reprogramming and preferentially leads to increased cell cycle gene expression and enhanced CM cycling [4]. H3K9me3’s function in cellular development, acting as a repressor of inappropriate lineage genes, and preserving cell integrity has been demonstrated in numerous studies. Diverse roles for H3K9me3 have been identified in regulating apoptosis [35], autophagy [36], development [37], DNA repair [38, 39], self-renewal [40], and aging [41], among others. Although H3K9me3 itself is not specific to cell cycle genes, in CM H3K9me3 depletion could preferntially increase cell cycle gene expression through the disruption of specific inhibitory complexes that bind H3K9me3 or H3K9me3-adapter proteins.

We sought to test the combined impact of Hippo pathway and epigenetic manipulations, individually or in combination, on CM cell cycle activity in both *in vitro* and *in vivo* settings. Our data demonstrate that combining KDM4D overexpression with inhibition of the Hippo pathway significantly increases CM cell cycle activity compared to either single treatment alone. We found that the Hippo pathway and KDM4D activate distinct transcriptional pathways, which likely accounts for their different effects. Combinatorial targeting of epigenetic and transcriptional pathways is a potential novel strategy for myocardial regeneration.

## Materials and methods

### Animals

All animals were maintained and experiments performed in accordance with an approved Institutional Animal Care and Use Committee (IACUC protocol #4290–01), institutional guidelines at the University of Washington, and National Institute of Health Guide for the Care and Use of Laboratory Animals. The inducible KDM4D mouse model used in this study was generated previously in our lab [4]. Tet-responsive-KDM4D mice were bred to the inducible αMHC-tTA (KDM4D^Tg/+^; tet-off) or repressible αMHC-rTA (iKDM4D; tet-on) mice [42, 43]. The tet-off KDM4D mice were crossed with our αMHC-MycER model [44] to generate a triple-transgenic mouse model (MycER+KDM4D^Tg/+^) that constitutively expresses KDM4D in the absence of doxycycline, with Myc induced by tamoxifen specifically in CMs. To assess the potential effects on cardiac function as a results of genetic manupliation, transthoracic echocardiography was performed prior to the endpoint as described previously [4]. Both gender of the animals were used, as we found no significant difference in our previous study [4]. For NRVM cultures, time-pregnant Sprague Dawley outbred rats (TP19) were purchased from Harlan/ENVIGO and CMs isolated as described [45]. Neonatal rats were sacrificed by decapitation, and adult mice were sacrificed by isoflurane overdose.

### Virus, miRNA, and siRNA

The virus vectors Ad-CMV-GFP-h-KDM4D (Ad-KDM4D), Ad-CMV-GFP-h-c-Myc (Ad-Myc), Ad-CMV-GFP-h-control (Ad-GFP), AAV9-U6-sh-Sav1-eGFP (AAV9-Sav1-sh), and its control AAV9-U6-scramble-eGFP (AAV9-C-sh) were purchased from Vector Biolabs (Malvern, PA). The miRNA and siRNA used in this study, including hsa-miR-199a-3p mimic (miR-199), hsa-miR-590-3p mimic (miR-590), hsa-miR-302b-5p mimic/hsa-miR-302c-5p mimic (miR-302), miRIDIAN microRNA Mimic Negative Control #1 (miR-C), ON-TARGETplus SMARTpool Sav1 siRNA (si-Sav1), and ON-TARGETplus control siRNA (si-C), were purchased from Dharmacon Inc. FGF Basic Recombinant Human Protein (PHG6015), EGF Recombinant Human Protein (PHG0313), and Recombinant Human Oncostatin M (HEK-293-expressed) Protein (PHC5015) were purchased from Thermo Fisher (USA).

### Cell culture and transfections

NRVMs were isolated as described before. FACS and immunostaining of α-actinin results demonstrate that NRVM purity was more than 99% (S1A–S1C Fig). NRVMs were cultured and treated as shown in S1A Fig. In brief, NRVMs were plated on fibronectin-coated 24-well plates at a density of 1×10^5 per well. NRVMs were seeded and cultured with 500μL M199 culture medium (Medium 199 500ml, HEPEs 10mM, MEM Non-Essential Amino Acids 1×, glucose 1.75g, L-glutamine 2mM, Vitamin B12 2mg, and penicllin 50,000 units) with 10% FBS. After 24 hours of serum medium culture, the NRVMs were infected with Ad-KDM4D, Ad-Myc, or control Ad-GFP in MOI 100 and maintained in the serum-free M199 culture medium (S1D Fig). The medium was changed to 500 μL fresh serum-free M199 culture medium at 24 hours after virus infection. At 48 hours after virus infection, the NRVMs were transfected with either miRNA (25nM) or siRNA (25nM) using Lipofectamine^™^ 3000 acording to the manufacturer’s protocol (Life Technologies) and/or treated with FGF (100ng/ml), EGF (100ng/ml), and OSM (50ng/ml). The transfection efficiency was assayed using miR-Dy547 control demonstrating 90% cells were transfected (S1E Fig). The NRVMs were maintained for another 48 hours, and then switched to the 500 μL fresh serum-free medium with 5nM 5-Ethynyl-2’-deoxyuridine (EdU). After 24 hours of EdU incubation, the cells were fixed for immunofluorescent staining or trypsinized with 0.05% Trypsin-EDTA for counting total cell numbers. The NRVMs were cultured 6 days in total since H3K9me3 levels were lowest after 5 days of infection (S3 Fig).

### In vivo gene transfer

To exam the effect of KDM4D and Sav1 on CM cell cycle activity *in vivo*, adult (8-12weeks) iKDM4D mice were injected with AAV9-Sav1-sh or control virus (AAV9-GFP). A thoracotomy was performed and mice were given three intramyocardial injections using Hamilton syringe (50μl capacity) with a 33-gauge needle to deliver a total of 2×10^11^ viral genomes (30μl total volume delivered) into the apex of the left ventricle [25]. 24 hours after injection, the mice were treated with Doxycycline in rodent chow and EdU in drinking water (ad.lib) until the study endpoint. Two weeks after injections, the mice were sacrificed, and the hearts harvested for immunostaining and RNA extraction. Anesthesia was induced with 5% isoflurane and maintained at 1–3% during surgery. Mice were sacrificed by isoflurane overdose two weeks after injection and heart tissue was harvested immediately.

### Isolation of adult mouse ventricular myocytes

For RNA-seq, cardiomyocytes were isolated using Langendorff perfusion digestion as previously described [5]. 8 to 10 week old iKDM4D mice were injected with AAV9-Sav1-sh or control virus (AAV9-GFP). After two weeks of injection, the mice were intraperitoneally injected with 200μl of heprin (100 IU/mouse) before being anesthetize with Isoflurane. The hearts were harvested and perfused with a 37°C Ca^2+^- free Tyrodes buffer (126 mM NaCl, 5.4 mM KCl, 0.33 mM NaH_2_PO_4_, 1 mM MgCl_2_, 10 mM HEPES, 10 mM Glucose, 20 mM Taurine, pH = 7.4) supplemented with 25 μM blebbistatin (Toronto Research Chemicals Inc. and BOC Sciences) for 1–2min, and then enzymatically digested at 37°C with 0.35 U/ml Liberase TH (Roche) prepared with blebbistatin supplemented-Ca^2+^- free Tyrode’s buffer for 8–12min. Heart tissues were mechanically dissociated in ice-cold KB solution (20 mM KCl, 10 mM KH_2_PO_4_, 70 mM Potassium Glutamate, 1 mM MgCl_2_, 25 mM Glucose, 20 mM Taurine, 0.5 mM EGTA, 10 mM HEPES, 0.1% Albumin, pH = 7.4). The cell suspensions were passed through a 100 μm cell strainer to remove tissue debris and then purified by low-speed centrifugation (50×g for 1min) 3 times, resulting in ~ 90% pure ACMs.

### RNA-seq

Two independent samples from each group were used for RNA-seq. Library preparation and sequencing was performed by commercial service (GENEWIZ). Sequence reads were trimmed to remove adapter sequences and nucleotides with poor quality using Trimmomatic v.0.36 [46]. The trimmed reads were mapped to the Mus musculus GRCm38 reference genome available on ENSEMBL using the STAR aligner v.2.5.2b [47]. Unique gene hit counts were calculated by using FeatureCounts from the Subread package v.1.5.2 [48]. The original read counts were normalized to adjust for various factors such as variations of sequencing yield between samples. These normalized read counts were used to accurately determine differentially expressed genes (DEGs). Using DESeq2, a comparison of gene expression between the groups of samples was performed [49]. The Wald test was used to generate p-values and log2 fold changes. Genes with an adjusted *p*-value < 0.05 and absolute log2 fold change > 1 were called as DEGs for each comparison. A gene ontology analysis was performed on the statistically significant set of genes by implementing the software GeneSCF v.1.1-p2 [50]. KEGG pathway analysis was performed by DAVID Bioinformatics Resources [51]. CiiiDER was used to scan transcription factors binding site on the promoter of DEGs [52]. Yap1/TEAD1-4 upregulated genes set was generated from Molecular Signatures Database v7.2 [53, 54].

### Histology and immunostaining

NRVMs or heart tissue sections were fixed with 4% PFA in PBS for 10 min, permeabilized with 0.2% Triton X-100 in PBS for 20min, and blocked with PBS containing 5% NDS (Normal Donkey Serum) for 1h at room temperature. For immunostaining, the cells were incubated overnight at 4°C with the following antibodies diluted in the blocking buffer: anti-cTnT (Thermo Scientific: MS-295-P) and Phalloidin (Thermo Scientific: R415) was used to identify CMs, Click-iT EdU Alexa Fluor 647 HCS Assay (Thermo Scientific: C10356) to identify the S phase of the cell cycle, anti-phospho-H3 (Thermo Scientific: PA5-17869) to identify the M phase of the cell cycle, anti-Aurora B antibody (Abcam: ab2254) to identify the cytokinesis phase of the cell cycle. The cells were then washed three times with PBS+5%FBS and stained for 45min at room temperature with secondary antibodies. This was followed by 5 min of DAPI (4′, 6-diamidino-2-phenylindole dihydrochloride). The cells were viewed under Nikon fluorescence microscope. To determine cross-sectional area (CSA) of cells, 10 μm thick heart slides were stained by Anti-wheat germ agglutinin (WGA). The CSA was determined by the region within the WGA staining boundary. At least 1000 myocytes per animal were analyzed using Image J’s “analyze particle” function. ACM measurements method was described before [4]. For ACM area and length measurements, isolated ACMs were fixed with 4% PFA, stained and imaged. The area and long-axis of hundreds of ACMs for each animal were manually traced and quantified using Image J’s “measure” function. We calculated CM volume using the formula: (mean ACM length x mean ACM transverse area (CSA)). CM number was estimated from the following formula: [mean Heart Volume (Heart mass/1.06, the density of muscle tissue [55]) / mean ACM volume x volume fraction of ACMs (the proportion of adult heart volume occupied by CMs)] [4].

### Quantitative real-time PCR

Total RNA was isolated using TRIzol^™^ Reagent (Invitrogen) and then purified using RNeasy Mini Kit (Qiagen). One microgram RNA was reverse transcribed using the High Capacity Reverse Transcription cDNA Synthesis Kit (Applied Biosystems). Quantitative PCR (qPCR) was performed using the Sybrgreen PCR master mix (Applied Biosystems) according to the manufacturer’s instructions and qPCR cycling was carried out on the AB7900. Primer sequences for qPCR are listed in S1 Table.

### Protein analysis

Western blots were performed by the SDS-PAGE electrophoresis. Total cell extracts were prepared and fractionated by gel electrophoresis and transferred to nitrocellulose membranes. The following primary antibodies were used: Anti-KDM4D (ab93694; Abcam), anti-H3K9me3 (ab8898; Abcam), anti-Sav1 (105105; Abcam), Yap1 (ab81183; Abcam), pYap1 (ab76252; Abcam), Myc (ab32072; Abcam), Anti-Histone H3 Antibody (07–690 from Sigma-Aldrich), GAPDH (ab181603; Abcam). Horseradish peroxidase anti-rabbit (ab205718; Abcam) was used as the secondary antibody. The membranes were initially cut according to the molecular weight size of proteins. The signal was detected using the super-signal-enhanced chemiluminescence system (Pierce).

### Statistical analysis

All *in vitro* experiments were carried out with at least n ≥3 biological replicates, unless otherwise specified. Animal or cells per group are identified in related figure legends. In experiments with multiple groups, analysis of variance (ANOVA) followed by Tukey’s test was used to compare group means. *P* value <0.05 was considered to represent a statistically significant difference. In all panels, numerical data are presented as mean ± SEM.

## Results

### Screening for growth factors and miRNAs that promote CM cycling

**Fig 1:**
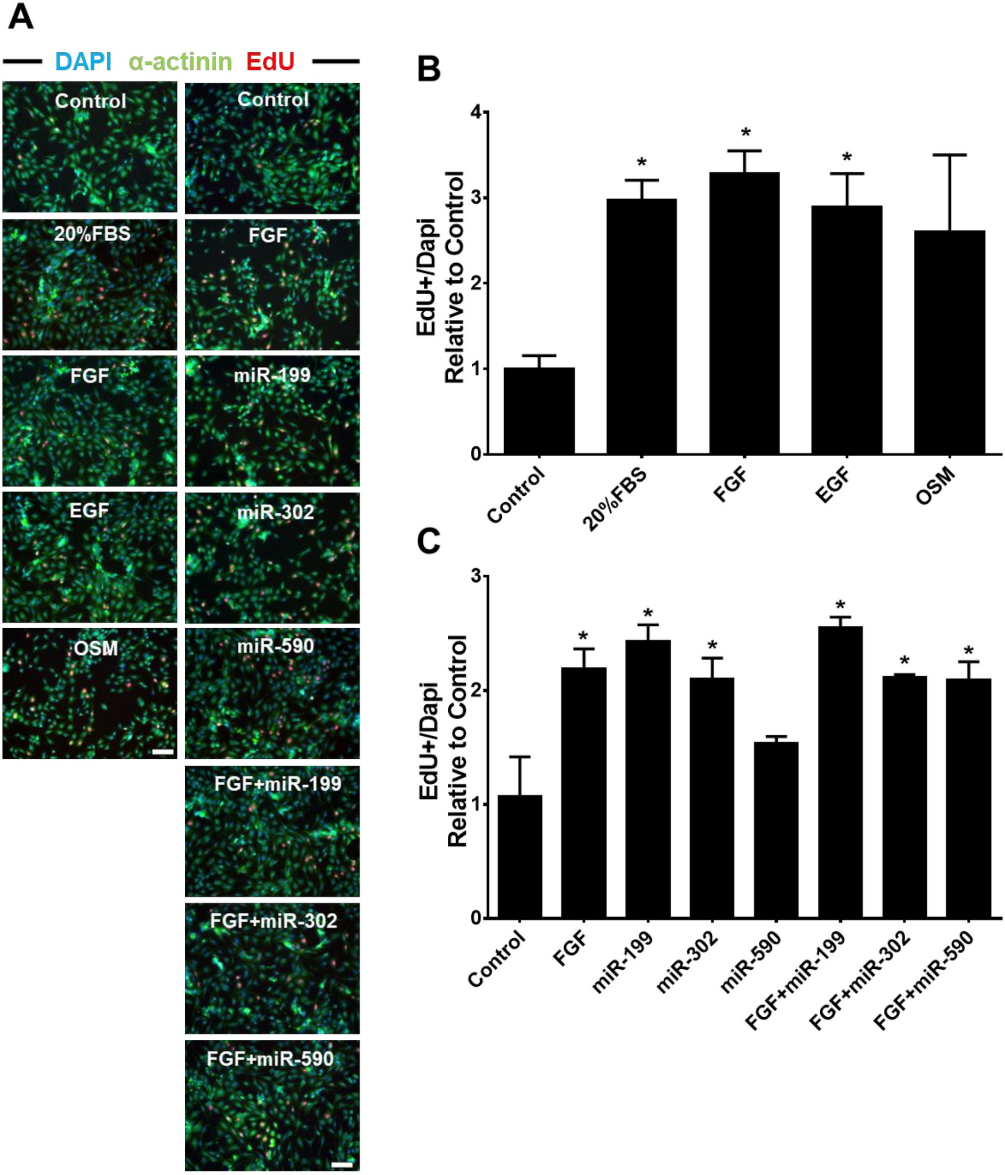
Screening of growth factors and miRNAs that promote CM cell cycle re-entry. (A) Representative photomicrographs showing EdU immunostaing after treating growth factors and/or miRNAs for 72 hours. Nuclei were stained by Dapi (blue), CMs by a-actinin (green) and cycling cells by EdU (red). Bar = 100μM. (B) Effects of indicated growth factors on CM EdU incorporation after 72 hours treatment in NRVMs. (C) Impact of overexpression of indicated miRNAs on CM EdU incorporation after 72 hours transfection in NRVMs. NRVMs were exposed to EdU for 24 hours. Statistics: Fold change of EdU^+^ NRVMs was normalized to control group; *p<0.05 in One-way ANOVA analysis followed by Tukeys’ Test; n = 3 for each group.

To identify factors that influence CM cell cycling, we screened growth factors and miRNAs that stimulate CM cycling using NRVMs [6, 8, 15, 16]. We found that all the growth factors tested increased EdU^+^ CM significantly except for OSM (~3-fold increases compared to control, *p*<0.05), but there were no differences in potency between factors (Fig 1A and 1B). Compared to the control group (treated with 25nM miR-C), both miR-199 and miR-302 treatment increased EdU^+^ CMs (~2.5-fold, *p*<0.05), while miR-590 had no significant effect. The effects of growth factors and miRs were not additive as stimulating NRVMs with FGF in addition to miRNAs did not further increase EdU^+^ cell number when compared to FGF or miRNA alone (Fig 1C).

### KDM4D and miR-199 preferentially impact different phases of cell cycle in vitro

**Fig 2:**
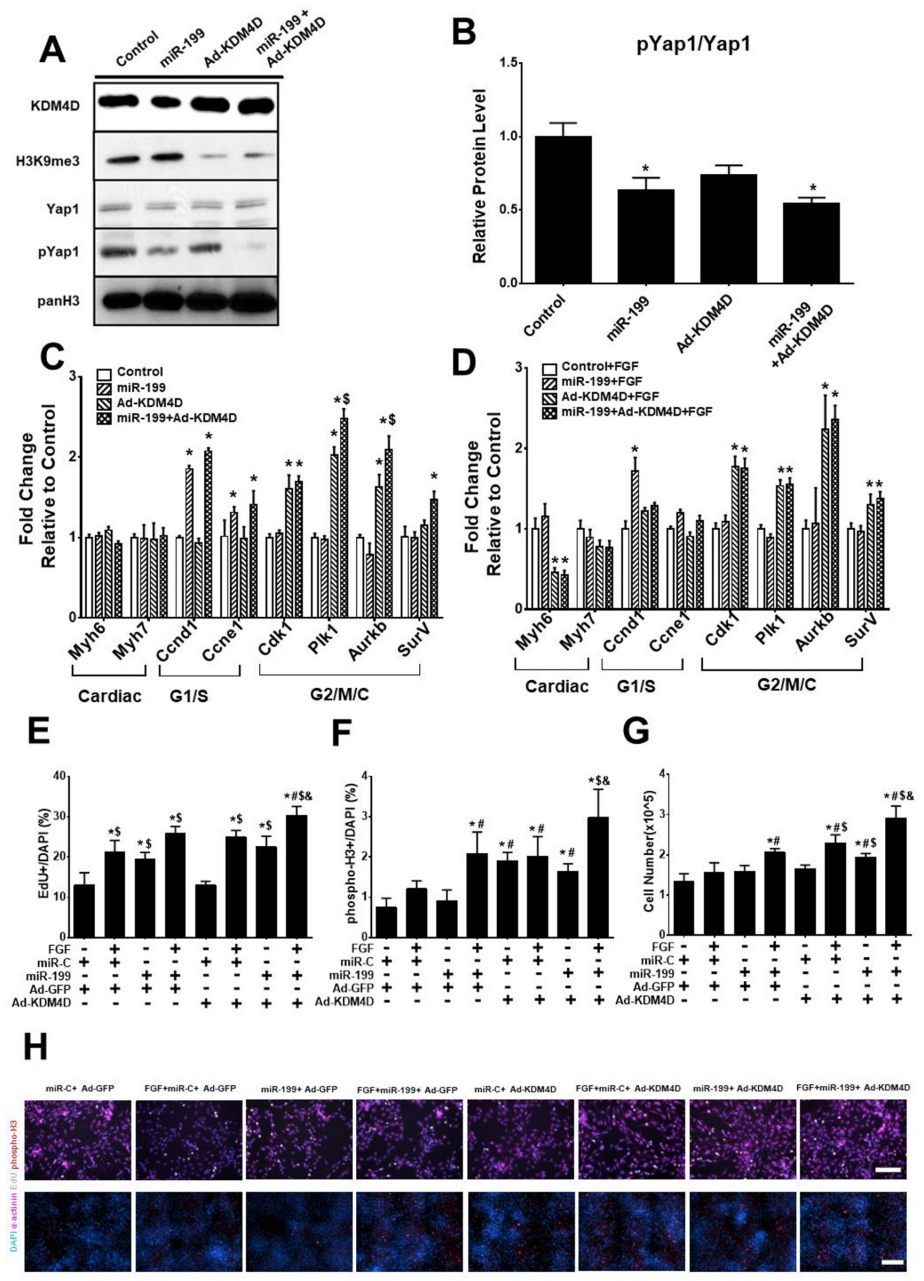
Effect of KDM4D and miR-199 on NRVM cell cycle activity. (A) Representative western blot of cell lysates from NRVMs treated with 5 days of Ad-KDM4D and 3 days of miR-199 (representative data from one of the three replicates). (B) Quantification of pYap1/Yap1 ratio from western blot. (C) RT-qPCR analysis shows the expression of cell cycle genes after 5 days of Ad-KDM4D and 3 days of miR-199 treatment. (D) The effect of FGF on KDM4D- and miR-199- stimulated cell cycle gene expression after 3 days (RT-qPCR assay). (E) EdU^+^ incorporation in NRVMs treated with or without Ad-KDM4D, miR-199, and FGF. (F) pH3^+^ immunostaining of NRVMs treated with indicated interventions. (G) Total cell number was measured by hemocytometer on NRVMs treated as indicated. (H) Representative photomicrographs showing EdU/phospho-H3 immunostaing after different treatments. EdU, bar = 100μM; phospho-H3, bar = 200μM. Statistics: * showed statistical significance at *p*<0.05 vs control (without miR-199, Ad-KDM4D, and FGF); # represented statistical significance at *p*<0.05 vs miR-199; $ mean statistical significance at *p*<0.05 vs Ad-KDM4D; & showed statistical significance at *p*<0.05 vs miR-199+Ad-KDM4D. One-way ANOVA followed by Tukeys’ Test; n = 3 for all the experiments.

To investigate the impact of KDM4D in addition to miRs on CM cell cycling, we treated NRVMs with Ad-KDM4D, miR-199, and miR-199+Ad-KDM4D. As expected, Ad-KDM4D reduced the levels of H3K9me3 (Fig 2A).

miR-199 decreased the proportion of pYap1/Yap1 in NRVMs as reported [25] (Fig 2A and 2B). Ad-KDM4D did not change Yap1 phosphyorlation (Fig 2B), suggesting different mechanisms of action (Fig 2A). Ad-KDM4D preferentially increased the expression of G2/M genes including Cyclin-dependent kinase 1 (*Cdk1*), polo-like kinase 1 (*Plk1*), Aurora Kinase B (*Aurkb*), and Survivin (*SurV*) expression, but did not significantly increase G1/S phase genes (Fig 2C). In contrast, miR-199 increased Cyclin D1 (*Ccnd1*) and Cyclin E1 (*Ccne1*) expression compared to control, but not *Cdk1*, *Plk1*, *Aurkb*, or *SurV* expression (Fig 2C). There was an additive increase in expression of Plk1 and Aurkb when KDM4D and miR-199 were combined (Fig 2C). We also tested for additive effects of FGF stimulation in combination with Ad-KDM4D or miR-199. FGF had no additive effect on Ad-KDM4D- or miR-199-induced cell cycle gene expression (Fig 2D). We next investigated the effect of Ad-KDM4D and miR-199 on CM cycling directly by examining EdU^+^ incorporation, phospho-H3^+^ (phospho histone H3) nuclei, and total cell number. FGF and miR-199 but not Ad-KDM4D increased EdU^+^ CMs compared to controls (Fig 2E). FGF+miR-199 or FGF+Ad-KDM4D treatment both increased EdU^+^ number, but not significantly more than FGF treatment alone (Fig 2E). In contrast, Ad-KDM4D increased pH3^+^ CMs as well as total cell number (Fig 2F and 2G).

### Additive effects of KDM4D overexpression and Hippo signaling inhibition on ACM cell cycle re-entry in vivo

**Fig 3:**
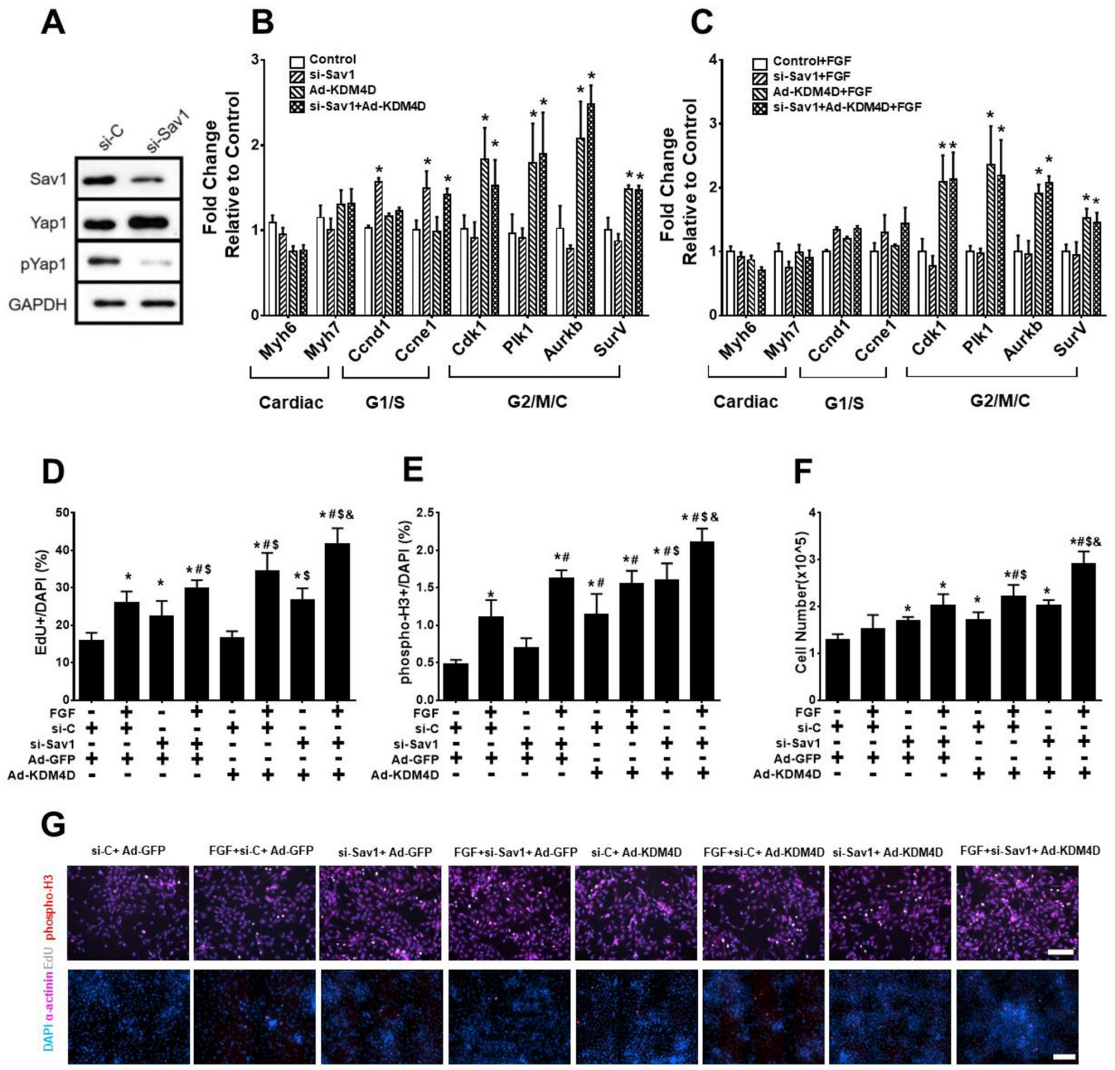
Effect of KDM4D and si-Sav1 on NRVM cell cycle activity. (A) Representative western blot of cell lysates from NRVMs after 3 days of Sav1 knockdown 199 (representative data from one of the three replicates). (B) Expression of cell cycle genes in Ad-KDM4D and si-Sav1 treated NRVMs. (C) Effects of adding FGF to Ad-KDM4D and si-Sav1 on cell cycle gene expression. (D) EdU^+^ incorporation in NRVMs treated with Ad-KDM4D and/or si-Sav1, and FGF. (E) phospho-H3^+^ immunostaining in NRVMs treated with or without Ad-KDM4D, si-Sav1, and FGF. (F) Total NRVM number after indicated treatments. (G) Representative photomicrographs showing EdU/phospho-H3 immunostaing after different treatments. EdU, bar = 100μM; phospho-H3, bar = 200μM. Statistics: * showed statistical significance at *p*<0.05 vs control (without si-Sav1, Ad-KDM4D, and FGF); # represented statistical significance at *p*<0.05 vs si-Sav1; $ mean statistical significance at *p*<0.05 vs Ad-KDM4D; & showed statistical significance at *p*<0.05 vs si-Sav1+Ad-KDM4D. One-way ANOVA followed by Tukeys’ Test; n = 3 for all the experiments.

This differential impact of Ad-KDM4D and miR-199 on cell cycle was unexpected. To confirm these results, we chose to test the effects of a direct regulator of the Hippo signaling pathway using siRNA to Sav1 (si-Sav1). As shown in Fig 3A, si-Sav1 reduced the ratio of pYap1 to Yap1, consistent with Yap1 activation. si-Sav1 and miR-199 had a similar effects on cell cycle gene expression in NRVMs (Fig 3B). si-Sav1 increased *Ccnd1* and *Ccne1* expression compared to control, but not *Cdk1*, *Plk1*, *Aurkb*, or *SurV* expression. FGF had no additive effect on the expression of these genes (Fig 3B). si-Sav1 treatment increased EdU^+^ CMs, but not phospho-H3^+^ CMs nor total cell number (Fig 3D–3F).

**Fig 4:**
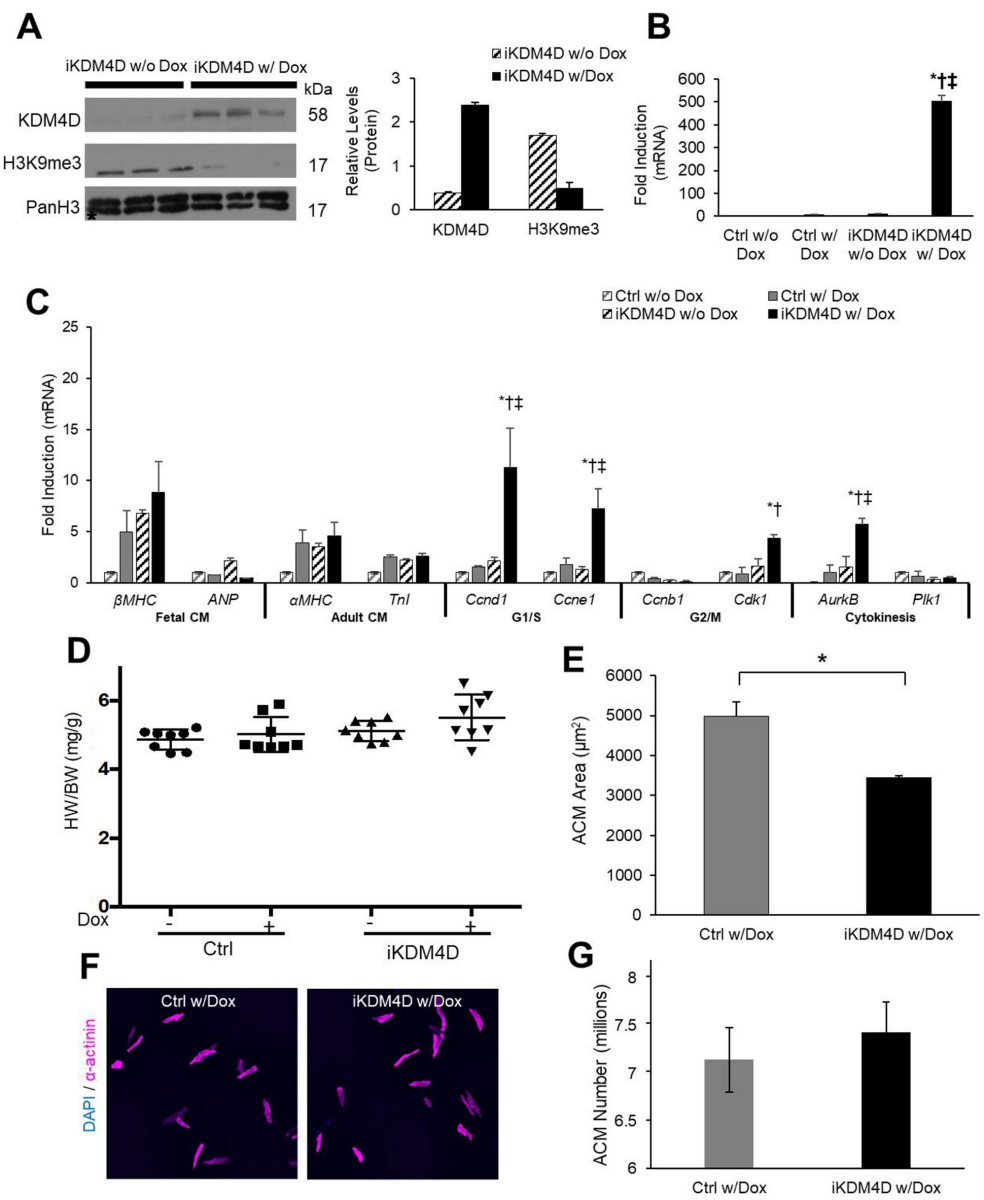
Inducible KDM4D mouse model. (A) Immunoblotting of ACM lysates (left) showing CM-specific KDM4D protein induction in iKDM4D hearts and depletion of H3K9me3 with two weeks of induction in ACMs. Densitometry analysis (right) shows KDM4D, and H3K9me3 levels relative to PanH3 control in iKDM4D CMs with (n = 3) and iKDM4D CMs without (n = 3) doxycycline induction. * *p*<0.05. (B) KDM4D transgene expression is robustly induced in iKDM4D ACMs fold induction vs. control (-Dox), expression normalized to Gapdh. (C) Expression of CM and cell cycle genes in isolated ACMs measured by qRT-PCR, fold induction vs. control (-Dox), expression normalized to Gapdh. (D) HW/BW quantification in uninduced control and iKDM4D mice (-Dox) and induced in control and iKDM4D (+Dox). (E, F, G) Quantification of ACM area (μm2) and ACM number, isolated 12-wk CMs control, and iKDM4D (+Dox). Sample Number: (A) iKDM4D (-Dox) = 3, iKDM4D (+Dox) = 3. (B) All groups = 8 (C) Control (+Dox) = 3, iKDM4D (+Dox) = 3 (D-G) ≥3 animals per group. Statistics: (A) Two-tailed t-test, iKDM4D (-Dox) vs iKDM4D (+Dox), *P < 0.05. (B) Two-way ANOVA/Tukey’s test, * p<0.05 vs. ctrl (-Dox), † p<0.05 vs. ctrl (+Dox), ‡ p<0.05 vs. iKDM4D (-Dox). (C) Two-way ANOVA/Tukey’s test, *P < 0.05 vs. ctrl (Dox), †P < 0.05 vs. iKDM4D (-Dox), ‡P < 0.05 vs. ctrl (+Dox). (D) Two-way ANOVA/Tukey’s test, P > 0.05 vs. Control (-Dox). (E) Two-tailed t-test, control (+Dox) vs. iKDM4D (+Dox), *P < 0.05.

To test if KDM4D overexpression and Sav1 knockdown also combinatorially promote ACM cell cycle re-entry *in vivo*, we firstly generated an inducible CM-specific KDM4D mouse model. CM-specific reverse tetracycline transactivator (rtTA) mice [56] were mated to a KDM4D tet-responder (tet) line (S3A Fig) [4]. The resulting mice (iKDM4D) displayed tightly regulated KDM4D gene expression in the heart with doxycycline treatment. Induction of KDM4D depleted H3K9me3 and up regulated cell cycle genes (Fig 4A–4C). Late cell cycle genes *Cdk1* and *AurkB* were up-regulated 4-fold and 6-fold, respectively (*p*<0.05). KDM4D induction did not result in a significant difference in HW/BW ratio in iKDM4D mice compared to control mice at two weeks (Fig 4D). However, CMs isolated from iKDM4D hearts had an average area 30% smaller than control CMs, (3437μm^2^ ± 55μm^2^ for iKDM4D and 4993μm^2^ ± 350μm^2^ for ctrl; *p*<0.01; Fig 4E). suggesting a total increase in the number of CMs. Consistent with this finding, estimated ACM number from iKDM4D hearts demonstrated a trend increase compared to control CMs (Fig 4G).

**Fig 5:**
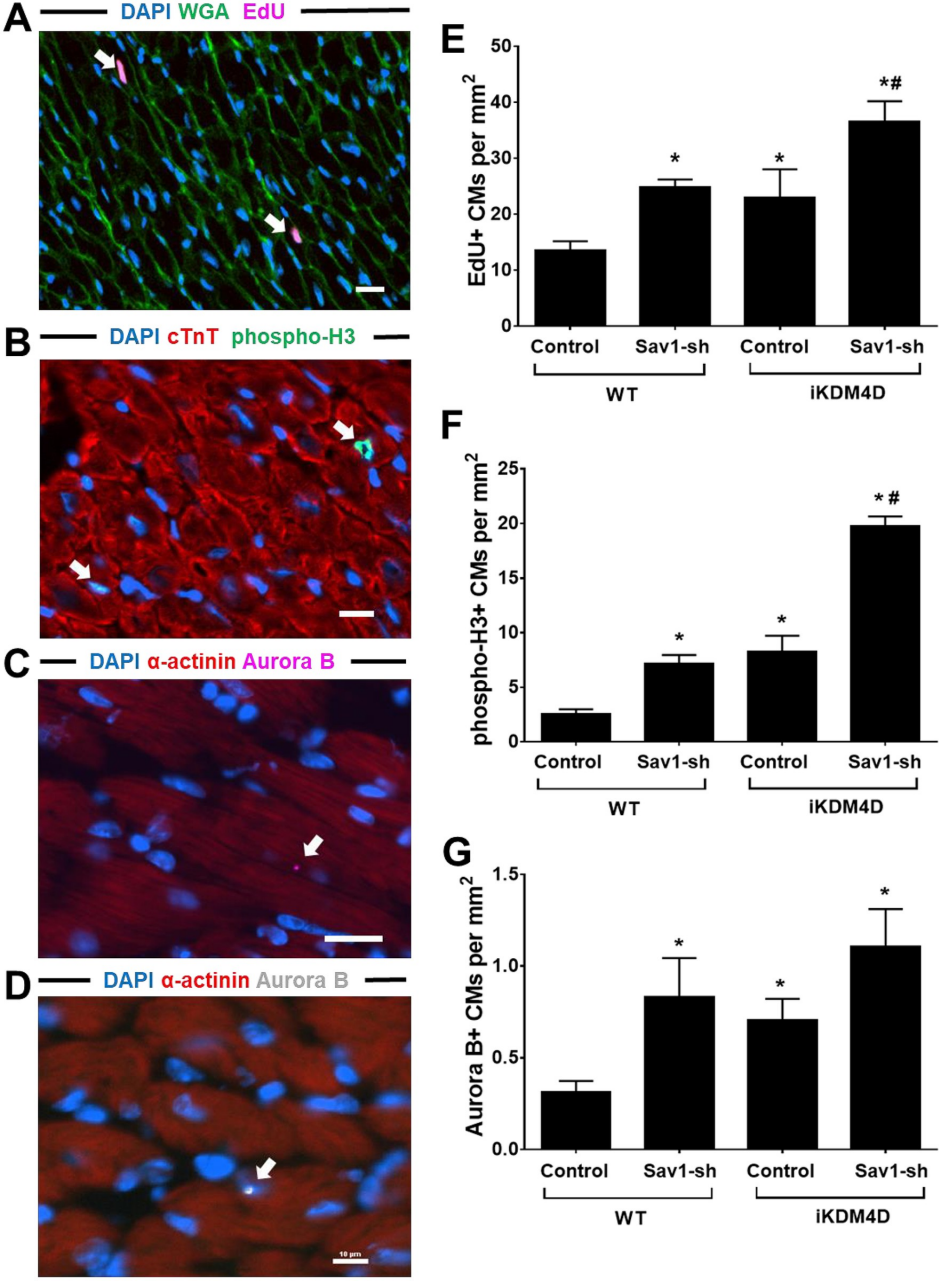
Additive effect of KDM4D overexpression and Sav1 knockdown on ACM cell cycle activity *in vivo*. (A, B, C, and D) Representative photomicrographs from iKDM4D+Sav1-sh hearts showing EdU, phospho-H3, and Aurora B immunostaing after KDM4D induction and Sav1 knockdown for 2 weeks. Nuclei were stained with DAPI (blue), cell borders with WGA (green), and cycling cells with EdU (magenta), white arrow points to the EdU^+^ CMs in (A). Nuclei were stained with DAPI (blue), CMs with cTnT (red), and cycling CM with phospho-H3 (green), white arrow points to the phospho-H3^+^ CMs in (B). (C) Aurora B (magenta, arrow) in a dividing CM. (D) Aurora B (white, arrow) in the nucleus of CMs. Nuclei were stained with DAPI (blue), CM actin with α-actinin (red), and cycling CM with Aurora B (magenta or white) in (C) and (D). Bar = 10μM. (E) Quantification of EdU^+^ ACMs in different groups. EdU^+^ CM number per mm^2^ is shown. (F) Quantification of phospho-H3^+^ ACMs in different group. Phospho-H3^+^ CM number per mm^2^ is shown. (G) Quantification of Aurora B^+^ ACMs in different group. Aurora B^+^ CM number per mm^2^ is shown. Statistics: n = 3 for each group. * showed statistical significance at *p*<0.05 vs control in WT group; # represented statistical significance at *p*<0.05 vs control in iKDM4D group. One-way ANOVA followed by Tukeys’ Test.

Next, we performed intramyocardial injections of AAV9-Sav1-sh to inhibit the Hippo signaling pathway in our iKDM4D mice. Whole heart sections displaying GFP+ CMs were chosen for imaging and cell counting (S3C Fig). In contrast to *in vitro* results, both iKDM4D and AAV9-Sav1-sh increased the number of EdU^+^, phospho-H3^+^, and Aurora B^+^ CMs at 14 days post-injection (Fig 5). When combined, iKDM4D and AAV9-Sav1-sh led to further increases in cell cycle activity including a 3-fold increase in DNA synthesis activity (Fig 5E), 7.8-fold increase in mitosis (Fig 5F), and ~3-fold increase in cytokinesis compared to wild-type control (Fig 5G).

### KDM4D and Hippo induce distinct transcriptional reprogramming in ACMs

**Fig 6:**
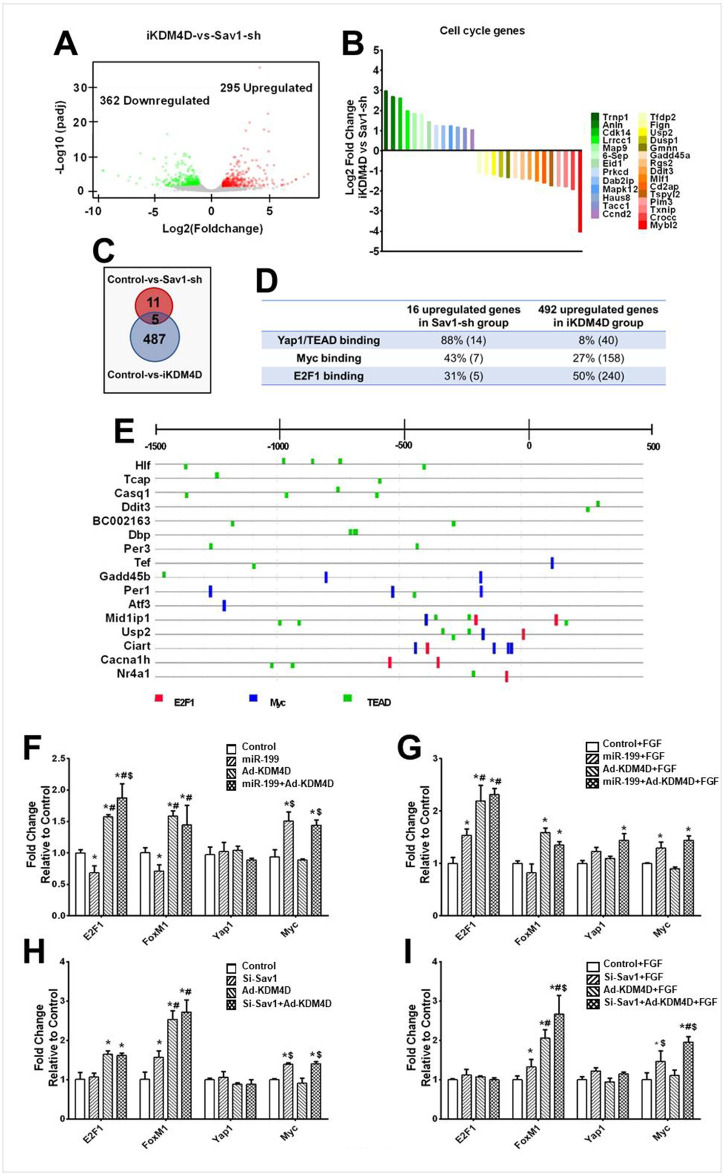
Transcriptional analysis of Sav1-sh and iKDM4D treated cardiac myocytes *in vivo*. (A) The global transcriptional change in the iKDM4D groups compared with Sav1-sh was visualized by a volcano plot. Each data point in the scatter plot represents a gene. The log2 fold change of each gene is represented on the x-axis and the log10 of its adjusted p-value is on the y-axis. Genes with an adjusted p-value less than 0.05 and a log2 fold change greater than 1 represent upregulated genes (red dots). Genes with an adjusted p-value less than 0.05 and a log2 fold change less than -1 represent downregulated genes (green dots). (B) Fold change of 28 cell cycle genes in iKDM4D group compared to Sav1-sh. (C) Summary of upregulated genes in Sav1-sh and iKDM4D group compared to control group. (D) Summary of promoter binding site analysis on upregulated genes in Sav1-sh and iKDM4D groups. Percentage of genes for each binding site are shown. The number in parenthesis represents the total number of genes. (E) Representative promoter binding site analysis. List promoters (-1500bp-500bp) of 16 upregulated genes in Sav1-sh group compared to control group. E2F1 binding site (red); Myc binding site (blue); TEAD binding site (green). (F, G, H, I) Expression of common cell cycle transcription factors after indicated treatments. Statistics: n = 3 for each group. * showed statistical significance at *p*<0.05 vs control (without miR-199, Ad-KDM4D, and FGF); # represented statistical significance at *p*<0.05 vs miR-199; $ mean statistical significance at *p*<0.05 vs Ad-KDM4D. One-way ANOVA followed by Tukeys’ Test.

To begin to understand the potential mechanisms underlying the differential effects we saw between KDM4D and Hippo pathway inhibition, RNA-seq was performed on control (wildtype mice injected with AAV9-GFP), Sav1-sh (wildtype mice injected with AAV9-U6-Sav1-sh-GFP), and iKDM4D (iKDM4D mice injected with AAV9-GFP) (S4A Fig). The sequencing data can be accessed at NCBI under BioProject Accession PRJNA902101. We identified 295 upreguatled genes and 362 downregulated genes in iKDM4D group compared to Sav1-sh group (Fig 6A). Among those differential expressed genes, GO analysis identified 28 cell cycle genes (Fig 6B). There were 13 genes upregulated in the iKDM4D group of which 11 are involved in cell division, including Trnp [57], Anln [58], Lrrcc1 [59], Map9 [60], 6-Sep [61], Eid1 [62], Prkcd [63], Dab2ip [64], Mapk12 [65], Haus8 [66], and Tacc1 [67]. 15 genes were upregulated in the Sav1-sh group compared to iKDM4D group of which 10 genes are involved in G1/S phase, including Tfdp2 [68], Usp2 [69], Gadd45a [70], Rgs2 [71], Ddit3 [72], Tspyl2 [73], Pim3 [74], Txnip [75], Crocc [76] and Mybl2 [77]. These data confirm that KDM4D preferentially induced expression of genes regulating late (G2/M) phases of the cell cycle, while Sav1-sh preferentially up-regulated genes involved in G1/S phase.

We also identified 16 upregulated genes and 48 downregulated genes in the Sav1-sh group compared to the control, and 492 upregulated genes and 882 downregulated genes in iKDM4D group compared to the control (Fig 6C and S4B Fig). Only 5 genes, including Dbp, BC002163, Casq1, Cacna1h, and Hlf, were upregulated in both the iKDM4D and Sav1-sh groups. We analyzed the promoters of the 16 upregulated genes in the Sav1-sh group and found the 14 (88%) of the upregulated genes contained at least one TEAD binding site, the target of Yap1/TAZ, but only 5 of them (31%) contained E2F1 binding sites, the proposed effector of KDM4Ds effects [4] (Fig 6D and 6E). We explored the promoters of 492 upregulated genes in the iKDM4D group and found 240 (50%) of genes contained at least one E2F1 binding site, but only 40 (8%) genes contained a TEAD binding site (Fig 6D).

To further explore the expression of transcription factors (TFs) involved in cell cycle we examined the levels of Yap1 and E2F1 along with their proposed targets Myc [78] and FoxM1 [79] in NRVMs in response to si-Sav1, miR-199, and KDM4D treatment. Both si-Sav1 and miR-199 expression resulted in no significant change in *E2F1*, *Foxm1*, and *Yap1* expression but a significant upregulation of *Myc* (*p*<0.05) (Fig 6F and 6H). In contrast, KDM4D overexpression increased *E2F1* and *FoxM1* expression (*p*<0.05) (Fig 6F and 6H). FGF had no additive effect over KDM4D, miR-199, or si-Sav1 on the expression of these TFs (Fig 6G and 6I). Consistent with these findings, the E2F1 promoter does not have any TEAD binding sites, but the Myc promoter contains four (S4C Fig). 7 (43%) of the promoters of the 16 Sav1-sh upregulated genes contained at least one Myc binding site (Fig 6D).

### KDM4D and Myc additively induce CM cell cycling but does not impact cardiac function

**Fig 7:**
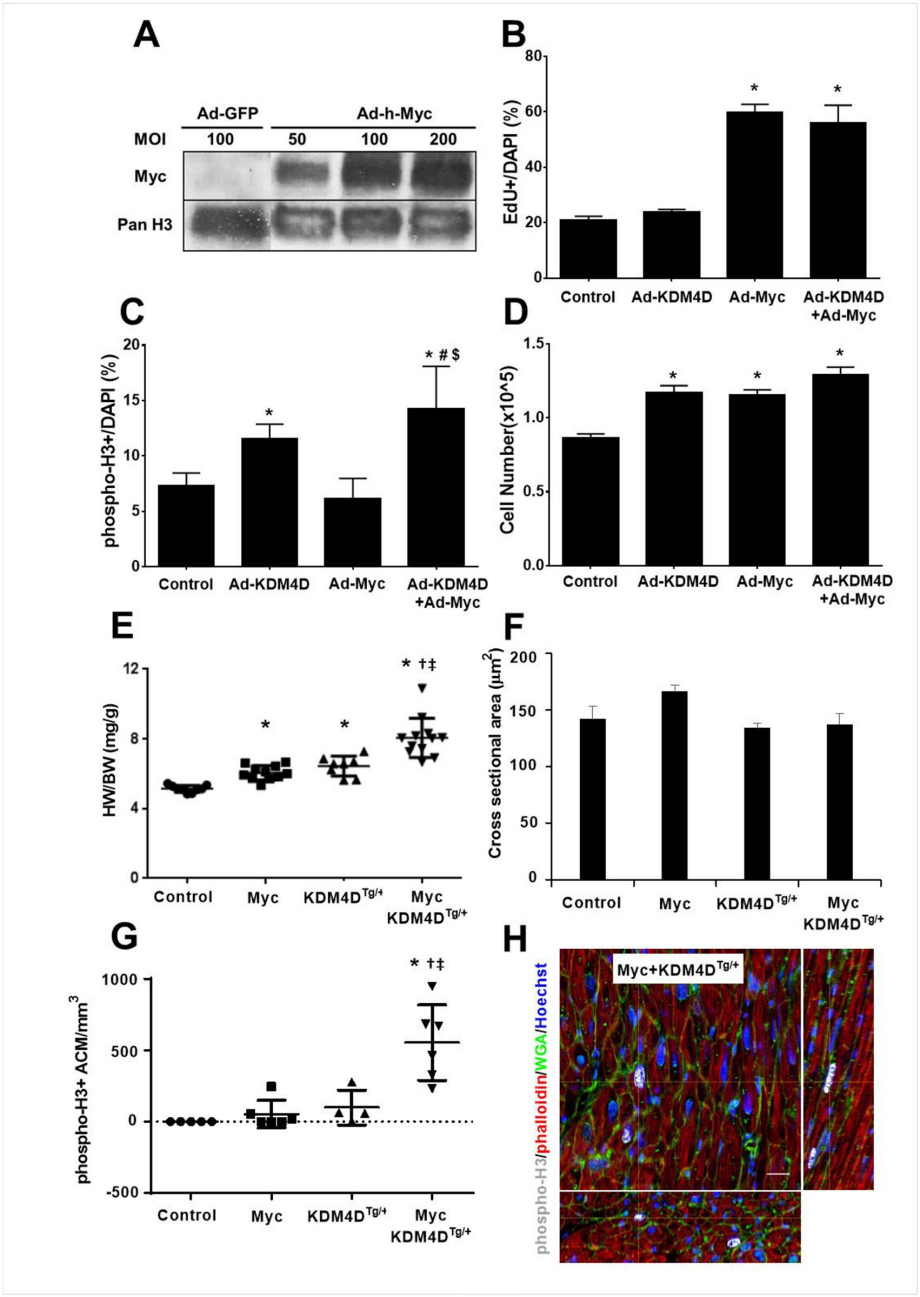
Additive effects of KDM4D and Myc expression on CM cell cycle activity. (A) Representative western blot after 5 days of Ad-Myc infection (MOI 50, 100, and 200) in NRVMs (representative data from one of the three replicates). (B, C, D) EdU^+^ incorporation, phospho-H3^+^ immunostaining, and total cell number in NRVMs treated with Ad-KDM4D or Ad-Myc for 5 days. Ad-GFP was used as control. (E, F, G) Quantification of HW/BW, ACM cross sectional area, and phospho-H3^+^ ACMs number at 10 weeks of MycER/KDM4D^Tg/+^ mice after one week of tamoxifen treatment. (H) Representative pictures showing phospho-H3 positive ACMs in transgenic MycER/KDM4D^Tg/+^ mouse model. Thick section imaging of adult BiTg heart showing XY plane and reconstructed 75 μm depth of XZ and YZ orthogonal planes. White crosshairs indicate position within all 3 planes, yellow arrows point to the phospho-H3^+^ ACMs, bar = 20 μm (n = 3 for each genotype). Statistics: *p< 0.05 vs Control, #*p*<0.05 vs Ad-KDM4D, $*p*<0.05 vs Ad-Myc, †*p*< 0.05 vs MycER, ‡*p*< 0.05 vs KDM4D^Tg/+^; One-way ANOVA followed by Tukeys’ Test.

Since both miR-199 and si-Sav1 upregulated *Myc* expression in addition to decreasing pYap1 in NRVMs, we tested whether the combination of KDM4D and Myc also have an additive effect on CM cell cycling. In NRVMs, Ad-Myc and Ad-KDM4D+Ad-Myc treatments increased Myc expression leading to a 3-fold enhancement of EdU^+^ CMs (Fig 7A and 7B). Ad-KDM4D treated NRVMs exhibited a ~2-fold increase in pH3^+^ CMs, (Fig 7C). Ad-KDM4D, Ad-Myc, and Ad-KDM4D+Ad-Myc all increased total cell number after 6 days of culture compared to the control (Fig 7D).

To test the effects of KDM4D and Myc *in vivo*, we generated a triple-transgenic mouse model (MycER+KDM4D^Tg/+^). In this model, KDM4D is constitutively expressed in CM while Myc is inducible activated in CM after tamoxifen treatment. In MycER or KDM4D^Tg/+^ transgenic mice (10-week old mice that received tamoxifen starting at 9 weeks), HW/BW ratio was increased 17% and 25% respectively compared to wild-type littermates (*p*<0.05), but dual expression of MycER and KDM4D had a ~1.6-fold increase in heart mass compared to control (*p*<0.05; Fig 7E). CM size was similar in these mice regardless of MycER or KDM4D expression (Fig 7F) suggesting that the increase in heart mass was due to increased CM number. This was supported by the finding of increased phospho-H3^+^ CMs in MycER+KDM4D^Tg/+^ hearts (Fig 7G and 7H). Functional analysis of MycER, KDM4D^Tg/+^, and MycER+KDM4D^Tg/+^ hearts by echocardiography confirmed a significant increase in LV mass compared to wild-type littermates; but no difference in HR (Heart Rate), EF (Ejection Fraction), FS (Fractional shortening), CO (Cardiac Output), and LVEDD (Left Ventricular End-Diastolic Dimension) among the different mouse models and control mice (Table 1).

**Table 1:** Cardiac function in MycER+KDM4D^Tg/+^ triple-transgenic mice.

	Control	Myc	KDM4D^Tg/+^	Myc + KDM4D^Tg/+^
**HR (*BPM*)**	**422 ± 9**	**451 ± 10**	**427 ± 10**	**424 ± 11**
**EF (*%*)**	**76.7 ± 2.1**	**81 ± 1.5**	**74.1 ± 1.5**	**76.5 ± 2.2**
**FS (*%*)**	**44.1 ± 1.9**	**48.2 ± 1.5**	**41.9 ± 1.4**	**44.1 ± 2**
**CO (*mL/min*)**	**10.7 ± 1**	**10.1 ± 0.6**	**12.5 ± 1.1**	**10.7 ± 1.2**
**LVEDD(mm)**	**2.92 ± 0.13**	**2.72 ± 0.08**	**3.13 ± 0.11**	**2.91 ± 0.14**
**LV mass (mg)**	**98 ± 7**	**122 ± 9**	**142 ± 7** *	**165 ± 13** * ^†^ ^‡^

Echocardiography results in 10-week old mice after 1 week of tamoxifen treatment. HR: Heart Rate, EF: Ejection Fraction, CO: Cardiac Output, LVEDD: Left Ventricular End-Diastolic Dimension, LV Mass: Left Ventricular Mass. Mean and SEM values are shown. *Sample Number*: Contol = 6, Myc = 8, KDM4D^Tg/+^ = 6, MycER+KDM4D^Tg/+^ = 7. *Statistics*: One-way ANOVA/Tukey’s test,

* *P*<0.05 vs Control,

^†^ *P*<0.05 vs Myc

^‡^ *P*<0.05 vs KDM4D^Tg/+^.

## Discussion

Previous studies in our lab revealed that although KDM4D reexpression induced cycling in transgenic hearts, the overall the magnitude was still relatively low and cell cycle gene expression was less than that seen in fetal CMs where proliferation normally occurs [4]. Thus, we hypothesized that in addition to depletion of the negative regulator H3K9me3, a stimulatory signal may also be required for more robust ACM cell cycling. Enhanced Yap1 activation through inhibition of Hippo signaling similarly induces ACM cell cycle reentry, but the level was also low [27, 80]. Given that KDM4D had no effect on Yap1 levels or phosphorylation status in our previous studies, we hypothesized that the Hippo signaling and epigenetic manipulation through KDM4D could have distinct mechanisms of action and therefore might have additive effects on promoting ACM cell cycle re-entry. To test this hypothesis, combinatinally overexpressed KDM4D and miR-199 or si-Sav1 and found that the combination potentiates CM cell cycle activity both *in vitro* and *in vivo*. Our data suggested that the Yap1 preferentially upregulates *Myc* expression while KDM4D increased E2F1 and FoxM1 expression, leading to CM cell cycle re-entry. Our ability to see these selective effects was replicated *in vivo*. The differential effects of these factors using this in vitro model versus the in vivo effect may related to the different developmental stage of NRVMs versus ACMs but the in vivo results in this study with KDM4D and Myc are similar to our previous studies [4, 44]. This difference may be also related to the shorter temporal exposure of NRVMs to these factors resulting in less secondary effects and the impact of the culture conditions we used [81]. We used serum free conditions to promote maturation of NRVMs.

miR-199 overexpression in CMs increases cell cycle genes expression [15] and decreases Hippo pathway genes, such as coffin 2 and TAOK1 [18, 26]. The ability of Hippo signaling and Yap/TAZ to preferentially promote G1/S transition has at least been implied by studies in nonmyocytes where it led to increased expression of *Ccne1* and *Cdc6* [82]. In endothelial cells, Yap1 is required for S-phase entry and knockdown of Yap1 led to accumulation of G1 phase cells and decreased the expression of G1/S phase genes [83]. Yap1 is also involved in Adriamycin-induced podocyte cell cycle re-entry by regulating *Ccnd1* and *Cdk4* expression thereby increasing entry into S phase [84]. In contrast, cardiac overexpression of KDM4D exhibits 5.8- to 21.4- fold increases of G2/M and cytokinesis genes, such as *Ccnb1*, *Cdk1*, *Plk1*, and *Aurk B* [4]. These results suggested that Hippo pathway inhibition preferentially activates the expression of genes that involved in G1/S phase, but KDM4D overexpression preferentially induces the expression of genes that involved in the late phase of cell cycle. Consistent with this, both the *in vitro* and *in vivo* studies demonstrated an additive effect of KDM4D overexpression and Hippo pathway inhibition on CM cell cycle re-entry.

To confirm this concept; namely, that KDM4D- and Hippo pathway- regulated CM cell cycle activity is through distinct mechanisms, RNA-seq was performed on CM samples from iKDM4D and Sav1-sh treated mice. Our RNA-Seq data indicated different transcriptional profiles between iKDM4D and Sav1-sh group. Consistent with *in vitro* qPCR data, KDM4D overexpression preferentially upregulated the G2/M phase genes, such as, Lrrcc1 [59], Map9 [60], and Dab2ip [64], which are involved in mitotic spindle formation. Mapk12 [65] and Haus8 [66] regulate the activity of Plk1 which plays an essential role in mitosis. Trnp [57], Anln [58], 6-Sep [61], Prkcd [63], and Tacc1 [67] also participate in the cell division. 10 of the 15 upregulated cell cycle genes in the Sav1-sh group are involved in the G1/S phase. Gadd45a [70] and Crocc [76] expression are higher in the G1 phase. Rgs2 [71], Ddit3 [72], Tspyl2 [73], and Txnip [75] are involved in the G0-G1 switch or G1 checkpoint. Tfdp2 [68], Usp2 [69], Pim3 [74], and Mybl2 [77] are also engaged in the G1/S phase of cell cycle. Regardless, our data suggest that the Hippo pathway and KDM4D regulate CM cell cycle re-entry through different mechanisms. In fact, KDM4D overexpression downregulated *TEAD3* expression, which is a transcriptional enhancer factor that plays a key role in the Hippo signaling pathway [85]. KDM4D overexpression upregulated *Patj* and *Mob1b* expression which play an important role in LATS1/2 and MST1/2 phosphorylation [86–88]. 14 upregulated genes in the Sav1-sh group contained TEAD binding sites, and 13 of them have been linked to cell cycling. 7 of the 13 cell cycle-related genes also contained canonical Myc binding sites. Among them, Mid1ip1, Usp2, Art3, and Nr4a1 impact the G1/S phase of the cell cycle in different cell types [89–92]. Myc can directly bind to the promoter regions of Nr4a1 and regulate its expression in hematopoietic stem cells [93]. Although studies have shown that Myc is a key molecular target of Yap1 in human cancer, this is the first report in CMs [78, 94]. Consistent with Myc as an effector of Yap1 in CM, overexpression of KDM4D and Myc in transgenic mice demonstrated marked cell cycle activity. Cardiac function was preserved, which has potential therapeutic relevance as persistent expression of miR-199 results in dysfunctiona and sudden arrhythmic death [26]. It is possible that miR-199 may alter the expression of additional genes or factors that alter electrophysiologic properties of CM in contrast to the KDM4D/ Myc combination.

In contrast to the Sav1-sh group, 51% of 492 promoters in KDM4D upregulated genes contained at least one E2F1 binding site, and only 9 genes are listed in the Yap1/TAZ/TEAD1-4 upregulated gene database [53, 54]. FoxM1, a G2/M-specific transcription factor that is a known target of E2F1 was also upregulated in iKDM4D hearts [95, 96]. Both E2Fs and FoxM1 play an important and well-established role in controlling the expression of genes important for the cell cycle, particularly in the control of gene expression at G2 phase of the cell cycle, encoding proteins known to function in mitosis [96, 97]. KEGG analysis of the 492 upregulated genes indicated that KDM4D regulated a number of genes involved in cell cycle-related pathways, including the ErbB signaling pathway, Wnt signaling pathway, and p53 signaling pathway (S4D Fig). ErbB signaling activation is required for G2 checkpoint activation in human breast cancer cells [98]. Inhibition of the ErbB signaling pathway induces G2/M arrest in gastric cancer cells [99]. Wnt/β-catenin signaling activity peaks during the G2/M phase [100], and inhibition of Wnt/β-catenin signaling leads to G2/M phase arrest [101]. Our data demonstrated that 9 downregulated genes in the iKDM4D group were enriched in p53 signaling pathways. Among them, Sfn (stratifin) and Gadd45g (growth arrest and DNA-damage-inducible 45 gamma) play a role in blocking G2/M transition through p53-dependent arrest [102, 103]. Other genes, such as Casp8, Serpine1, Shisa5, Thbs1, Mdm4, Igfbp3, and Cdkn1a, are all involved in p53-regulated apoptotic progression [104–109]. Thus, our data imply that KDM4D overexpression preferentially stimulates gene expression controlling the G2/M phase of the cell cycle through E2F1 activity. In addition to regulating E2F1 expression, KDM4D may also regulate E2F1 activity since protein lysine methyltransferases and demethylases can modify a specific lysine residue on non-histone substrates with one or more methyl moieties, such as E2F1 [110], which impacts the activity or subcellular localization of the substrate protein [111–114].

**Fig 8:**
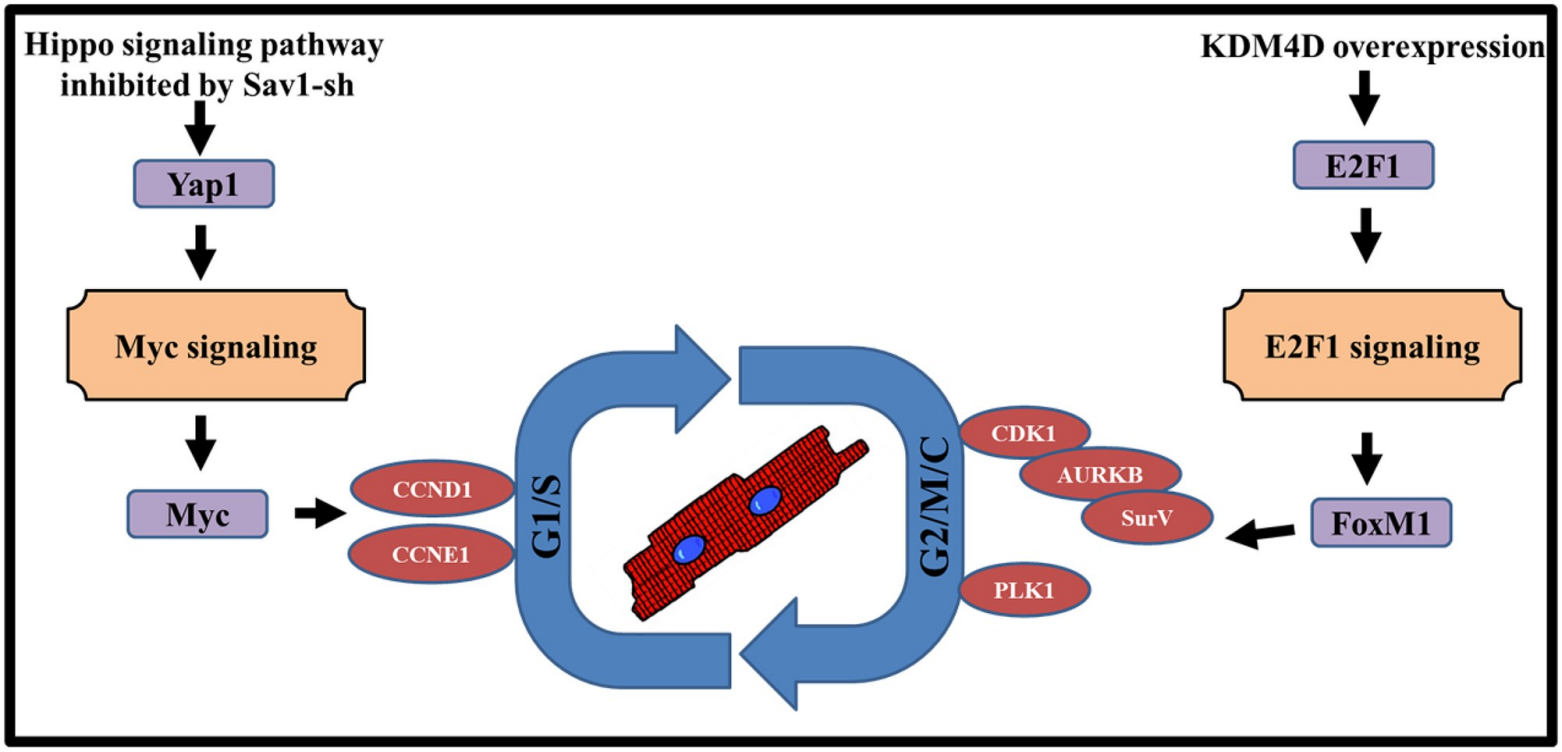
Proposed model for KDM4D and Hippo signaling pathway in the regulation of cell cycle activities. KDM4D preferentially induced expression of genes regulating late (G2/M) phases of the cell cycle by stimulating E2F1 and FoxM1 expression, while miR-199 or Hippo pathway inhibition preferentially up-regulated genes involved in G1/S phase by stimulating Myc expression.

In conclusion, KDM4D and miR-199/si-Sav1 combinationally promote CM cycling through distinct pathways (Fig 8). Si-Sav1 or miR-199 preferentionally induce G1/S phase cell cycle genes at least in part through activating Myc signaling pathway, while KDM4D promotes G2/M phase by regulating E2F1 and FoxM1 expression. Importantly, we confirmed our additive *in vitro* results *in vivo*, demonstrating these interventions also work in ACMs. Enhancing endogenous CM cell cycle activity to compensate for the lost CMs after injury may be a promising strategy to prevent the development of heart failure.

## Supporting information

S1 Fig NRVM purity, virus infection efficiency, and miRNA transfection efficiency. (A) Timeline showing protocol for NRVM in vitro study. (B) FACS results showing the purity of NRVM after one day of culture by staining cTnT and NKX2.5 protein. (C) Immunostaining of α-actinin showing the purity of NRVM after 5 days of culture. Blue color represents the nuclei and magenta color represents the α-actinin positive cells. (D) MOI selection by detecting GFP expression after 5 days of infection. (E) small RNA transfection efficiency was detected by miR-Dy547 after 3 days of transfection. Left panel was the representative picture. Red color represents the miR-Dy547 transfected cells. Right panel was the quantification of miR-Dy547 transfected cells. Data are shown as mean ± SEM (n = 3 independent experiments).
(TIF)

S2 Fig KDM4D expression level after Ad-KDM4D infection in NRVMs. (A) KDM4D expression level increased after 3 days of Ad-KDM4D infection detected by qPCR. Sample number = 1 for each treatment. (B) Timeline showing the protocol for KDM4D and H3K9me3 western blot analysis. (C) KDM4D and H3K9me3 protein expression level at different time point after Ad-KDM4D infection detected by western blot. Sample number = 1 for each treatment.
(TIF)

S3 Fig Inducible KDM4D mouse model construction, induction protocol, and model testing after myocardial injection of AAV9-Sav1-sh and AAV9-control. (**A**) Schematic showing breeding strategy resulting in iKDM4D mice, and KDM4D induction in BiTg CMs. (**B**) Timeline showing protocol for ACM-specific KDM4D expression and endpoints. (C) The whole heart scanning showing the myocardial injection efficiency after 2 weeks (representative image from one of the three animals in each group).
(TIF)

S4 Fig RNA-seq in iKDM4D mouse model. (A) The pipeline of the RNA-seq. (B) The global transcriptional change in the Sav1-sh and iKDM4D groups compared with control was visualized by a volcano plot. Each data point in the scatter plot represents a gene. The log2 fold change of each gene is represented on the x-axis and the log10 of its adjusted p-value is on the y-axis. Genes with an adjusted p-value less than 0.05 and a log2 fold change greater than 1 represent upregulated genes (red dots). Genes with an adjusted p-value less than 0.05 and a log2 fold change less than -1 represent downregulated genes (green dots). (C) Transcription factors binding site analysis on the common cell cycle transcription factors promoter. The promoter sequence was analyzed from -1500 to +500. Red bars represent E2F1 binding site, blue bars represent Myc binding site, and green bars represent TEAD binding site. (D) KEGG pathway analysis between control and iKDM4D by DAVID Bioinformatics Resources. The number of genes is represented on the x-axis and the KEGG pathways are listed on the y-axis.
(TIF)

S1 Table DEGs in iKDM4D group compared to Sav1-sh. (XLSX)

S2 Table Upregulated DEGs in group Sav1-sh compared to control. (XLSX)

S3 Table Upregulated DEGs in group iKDM4D compared to control. (XLSX)

S4 Table Upregulated DEGs in both Sav1-sh and iKDM4D. (XLSX)

S5 Table Primers used in this study. (XLSX)

S1 Raw images (PDF)

## Abbreviations

AAV: Adeno-associated viruses
Aurora B: Aurora kinase B
Ccnd1: Cyclin D1
Ccne1: Cyclin E1
Cdk1: Cyclin Dependent Kinase 1
cTnT: cardiac troponin T
E2F1: E2F transcription factor 1
EdU: 5-ethynyl-2’-deoxyuridine
FoxM1: Forkhead Box M1
GFP: green fluorescent protein
H3K9me3: Histone H3 lysine 9 trimethylation
KDM4D: Lysine Demethylase 4D
Myc: MYC Proto-Oncogene, BHLH Transcription Factor
Myh6: α-myosin heavy chain
Myh7: β- myosin heavy chain
NRVM: neonatal rat ventricular myocyte
phospho-H3: Phospho-Histone H3
Plk1: Polo Like Kinase 1
Sav1: Salvador Family WW Domain Containing Protein 1
SurV: survivin
Yap1: Yes-associated protein1
α-SA: α-sarcomeric actinin
