# Supplementary figures and images for: Hippo signaling and histone methylation control cardiomyocyte cell cycle re-entry through distinct transcriptional pathways
Source: PLoS One. 2023 Feb 13;18(2):e0281610. doi: 10.1371/journal.pone.0281610 (PMC9925018; doi:10.1371/journal.pone.0281610)

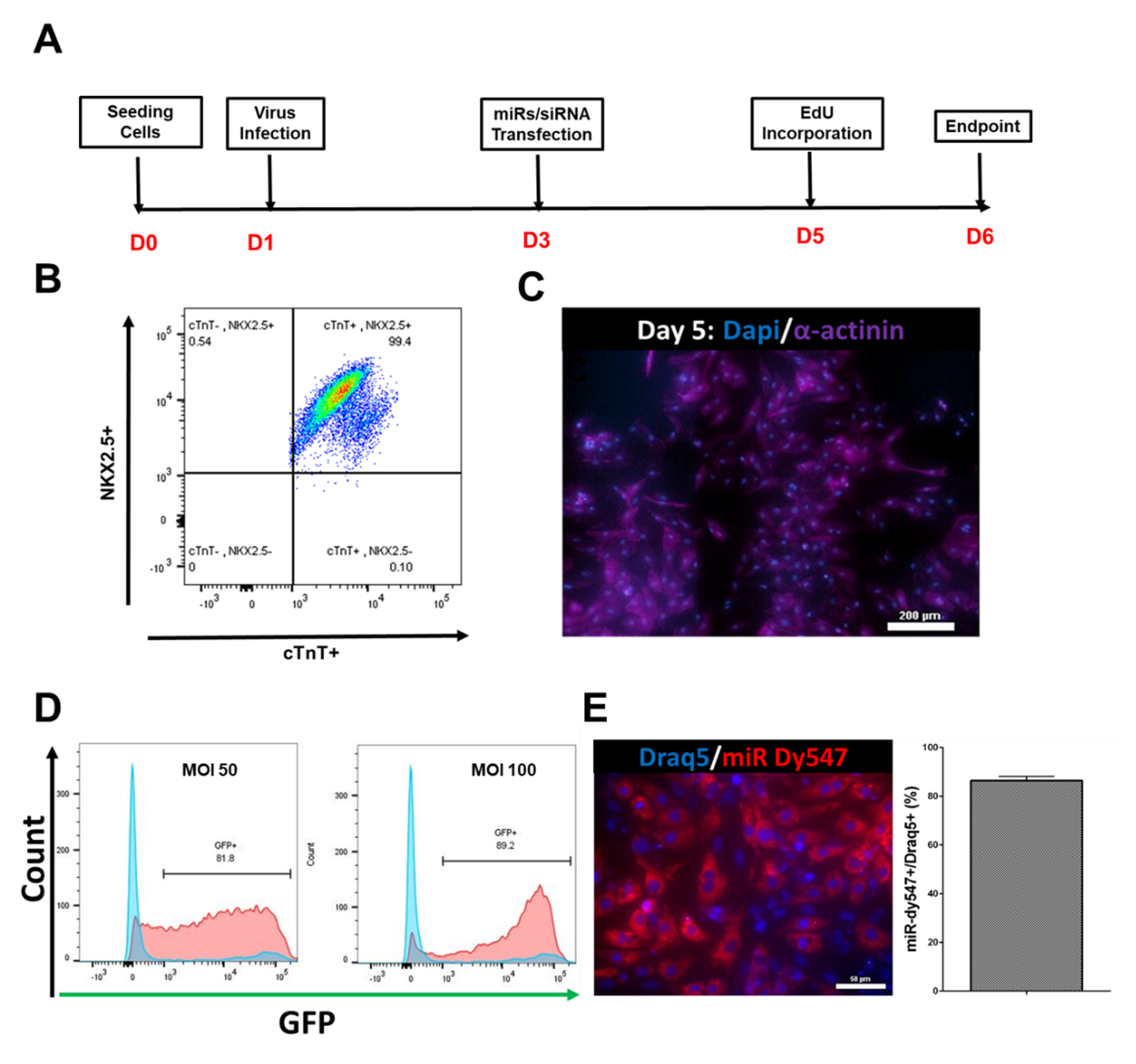

Supplement: S1 Fig — (A) Timeline showing protocol for NRVM in vitro study. (B) FACS results showing the purity of NRVM after one day of culture by staining cTnT and NKX2.5 protein. (C) Immunostaining of α-actinin showing the purity of NRVM after 5 days of culture. Blue color represents the nuclei and magenta color represents the α-actinin positive cells. (D) MOI selection by detecting GFP expression after 5 days of infection. (E) small RNA transfection efficiency was detected by miR-Dy547 after 3 days of transfection. Left panel was the representative picture. Red color represents the miR-Dy547 transfected cells. Right panel was the quantification of miR-Dy547 transfected cells. Data are shown as mean ± SEM (n = 3 independent experiments). (TIF) [file pone.0281610.s001.tif]

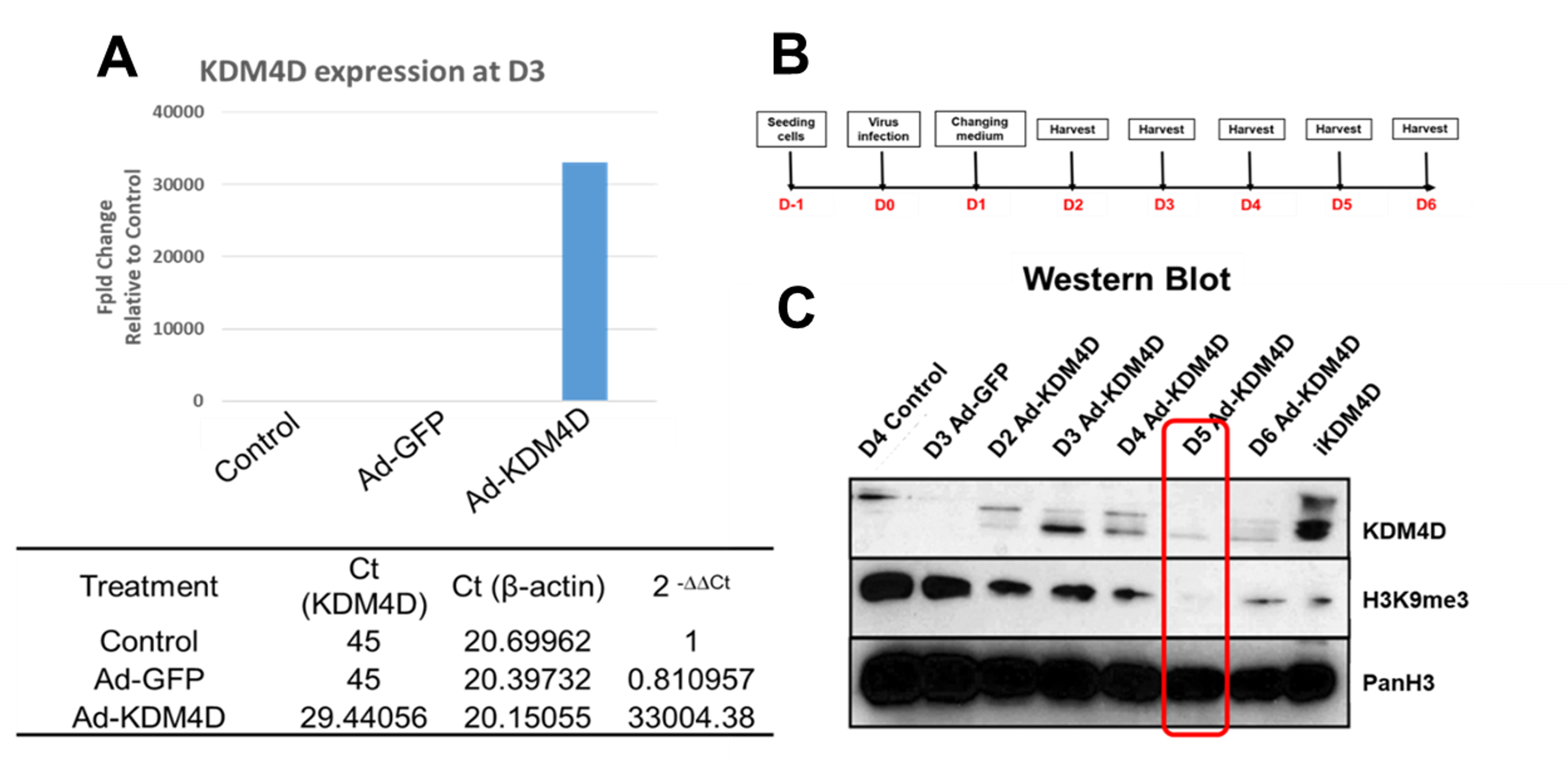

Supplement: S2 Fig — (A) KDM4D expression level increased after 3 days of Ad-KDM4D infection detected by qPCR. Sample number = 1 for each treatment. (B) Timeline showing the protocol for KDM4D and H3K9me3 western blot analysis. (C) KDM4D and H3K9me3 protein expression level at different time point after Ad-KDM4D infection detected by western blot. Sample number = 1 for each treatment. (TIF) [file pone.0281610.s002.tif]

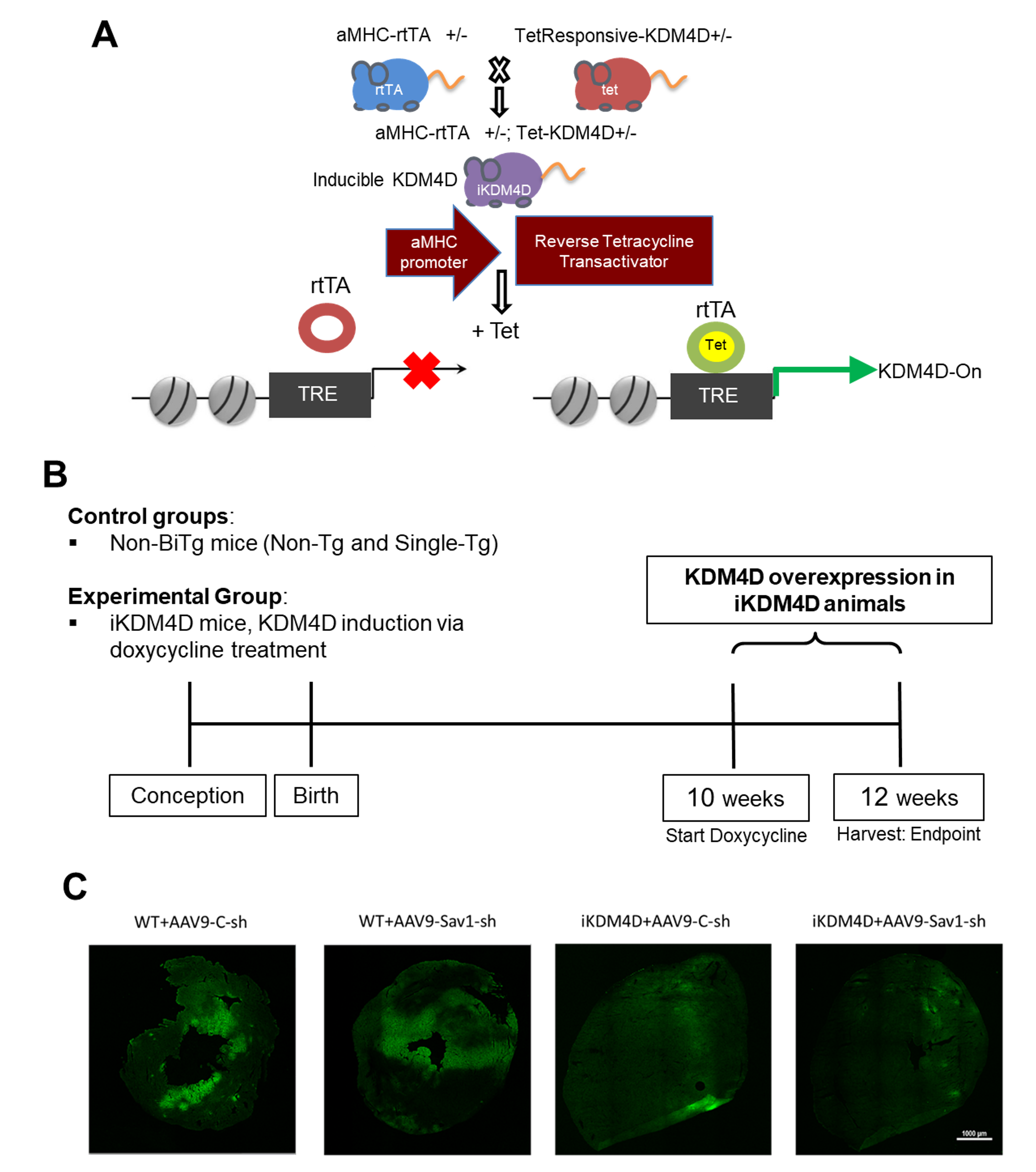

Supplement: S3 Fig — (A) Schematic showing breeding strategy resulting in iKDM4D mice, and KDM4D induction in BiTg CMs. (B) Timeline showing protocol for ACM-specific KDM4D expression and endpoints. (C) The whole heart scanning showing the myocardial injection efficiency after 2 weeks (representative image from one of the three animals in each group). (TIF) [file pone.0281610.s003.tif]

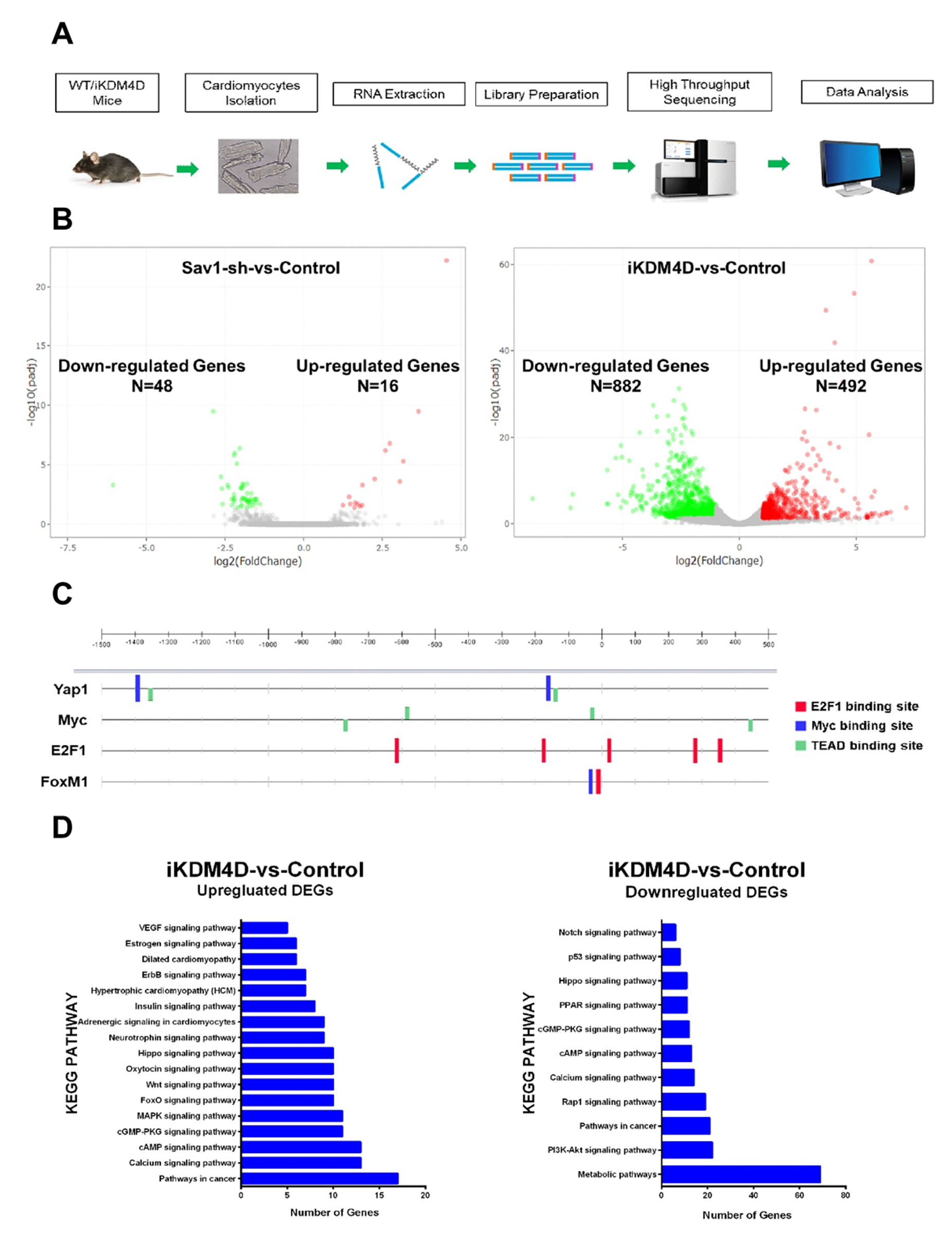

Supplement: S4 Fig — (A) The pipeline of the RNA-seq. (B) The global transcriptional change in the Sav1-sh and iKDM4D groups compared with control was visualized by a volcano plot. Each data point in the scatter plot represents a gene. The log2 fold change of each gene is represented on the x-axis and the log10 of its adjusted p-value is on the y-axis. Genes with an adjusted p-value less than 0.05 and a log2 fold change greater than 1 represent upregulated genes (red dots). Genes with an adjusted p-value less than 0.05 and a log2 fold change less than -1 represent downregulated genes (green dots). (C) Transcription factors binding site analysis on the common cell cycle transcription factors promoter. The promoter sequence was analyzed from -1500 to +500. Red bars represent E2F1 binding site, blue bars represent Myc binding site, and green bars represent TEAD binding site. (D) KEGG pathway analysis between control and iKDM4D by DAVID Bioinformatics Resources. The number of genes is represented on the x-axis and the KEGG pathways are listed on the y-axis. (TIF) [file pone.0281610.s004.tif]

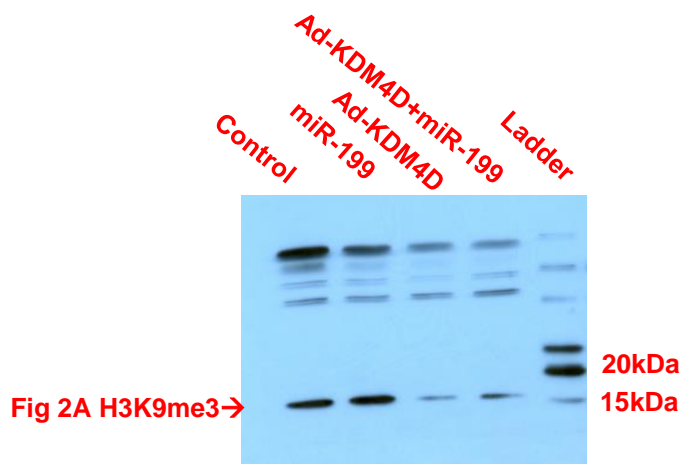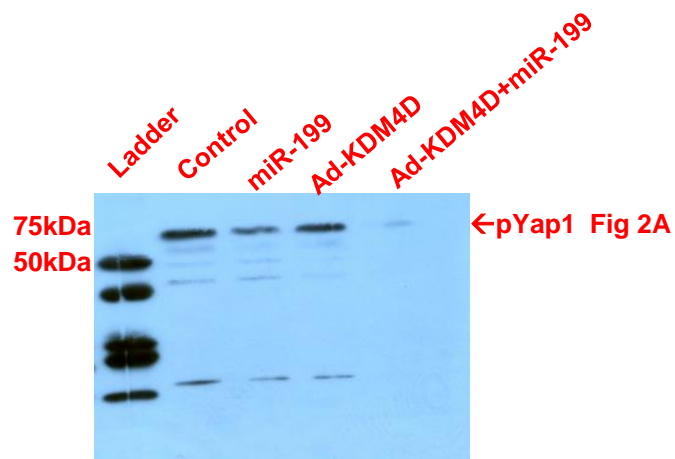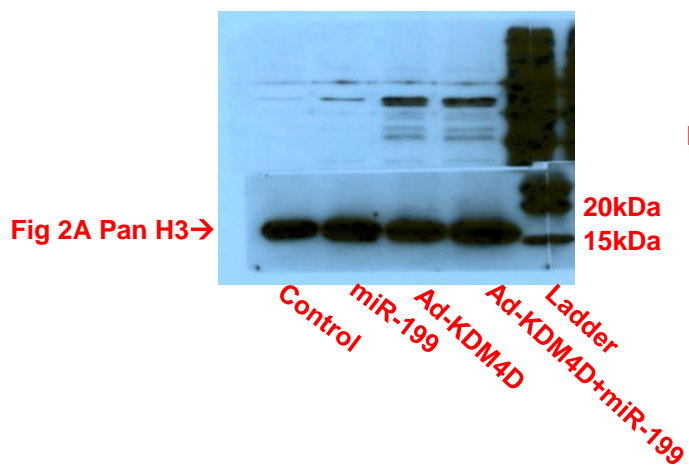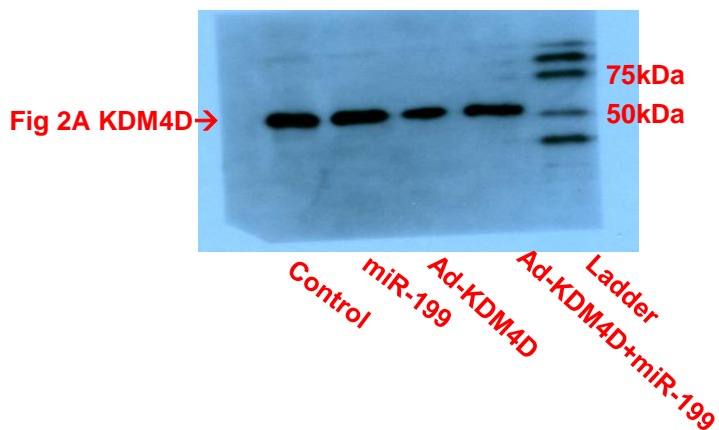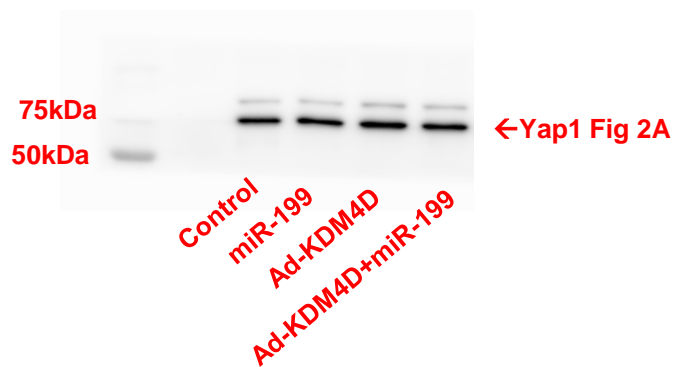

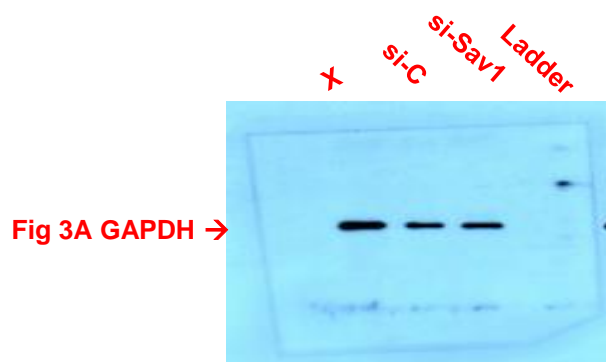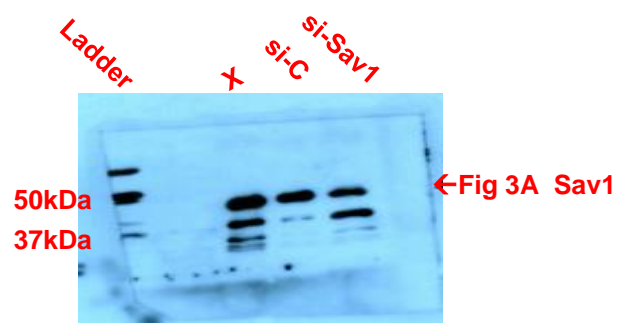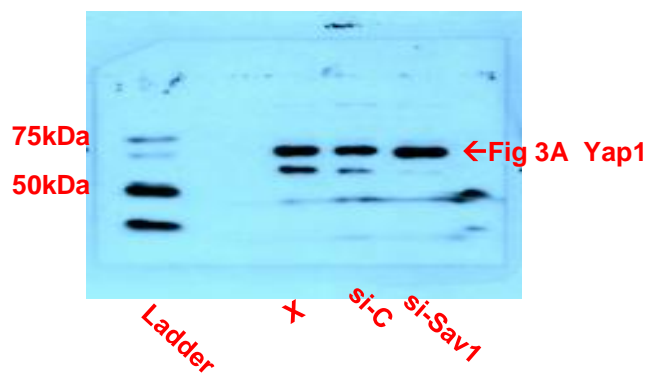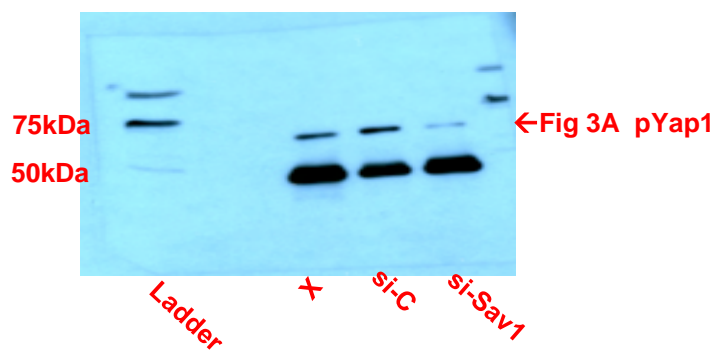

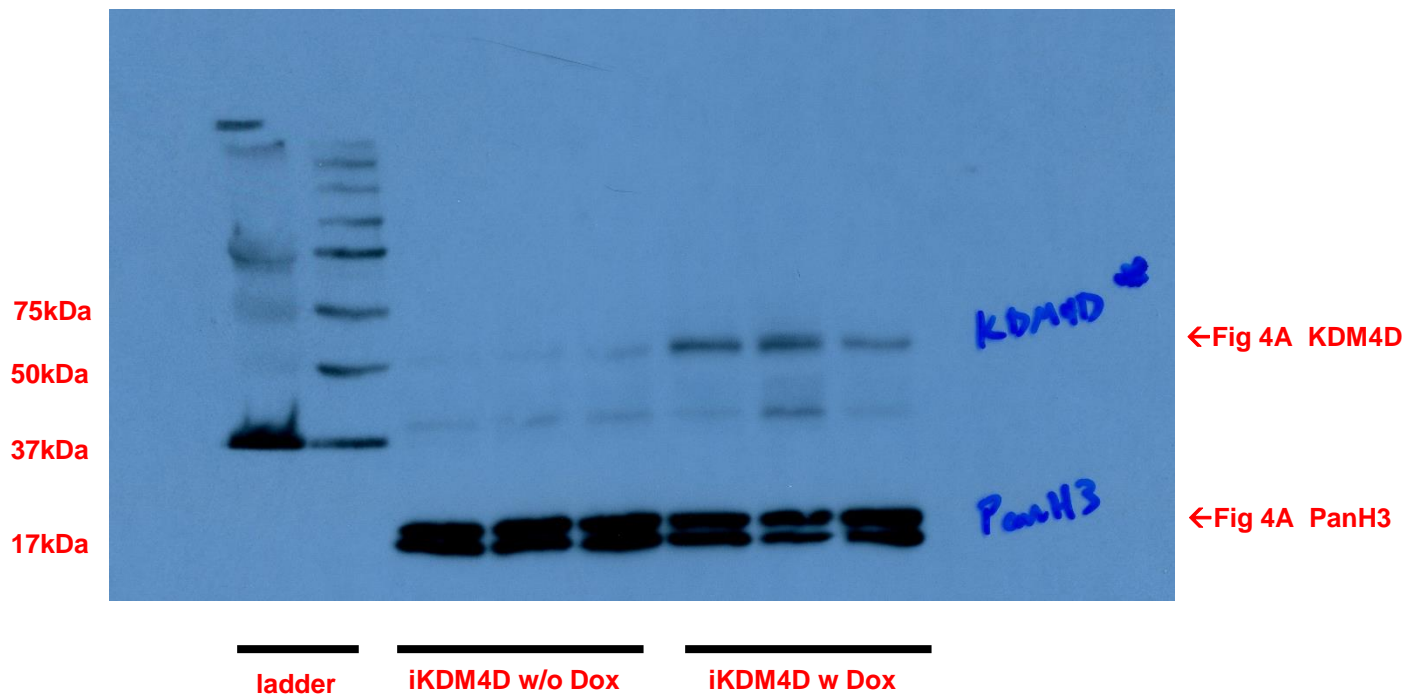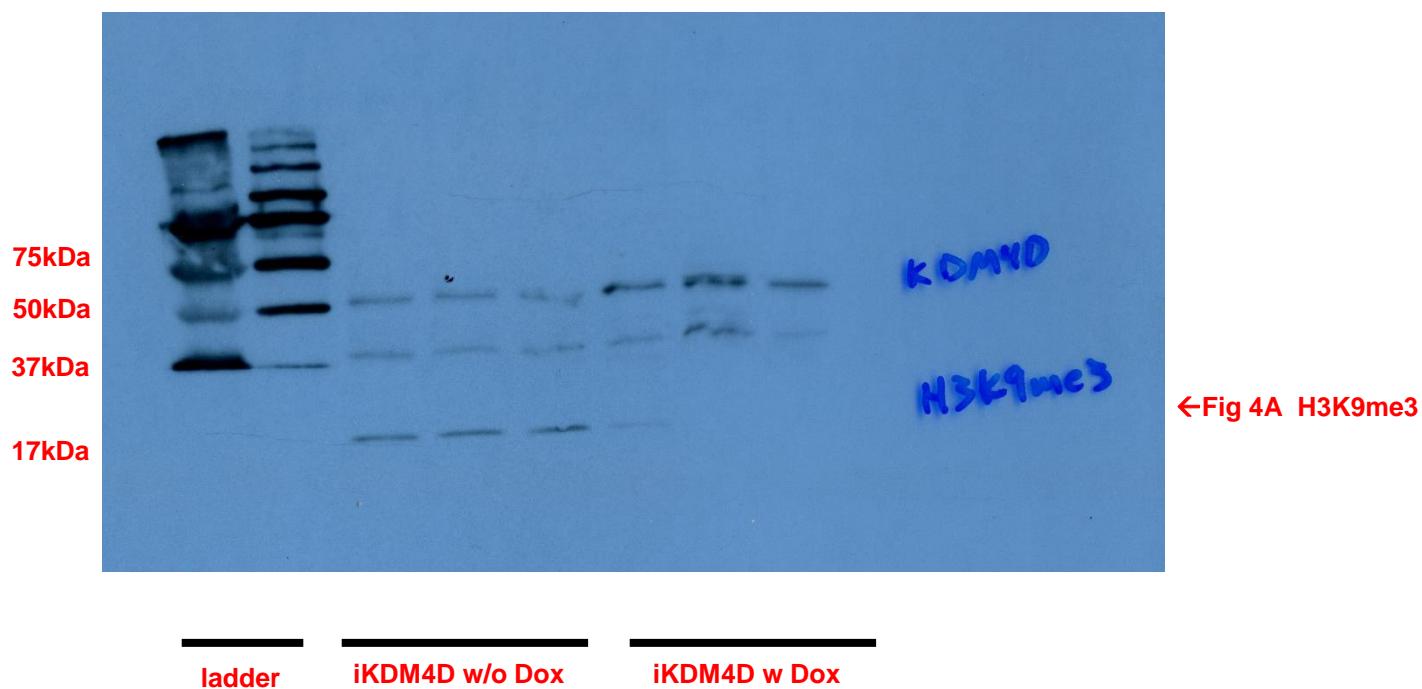

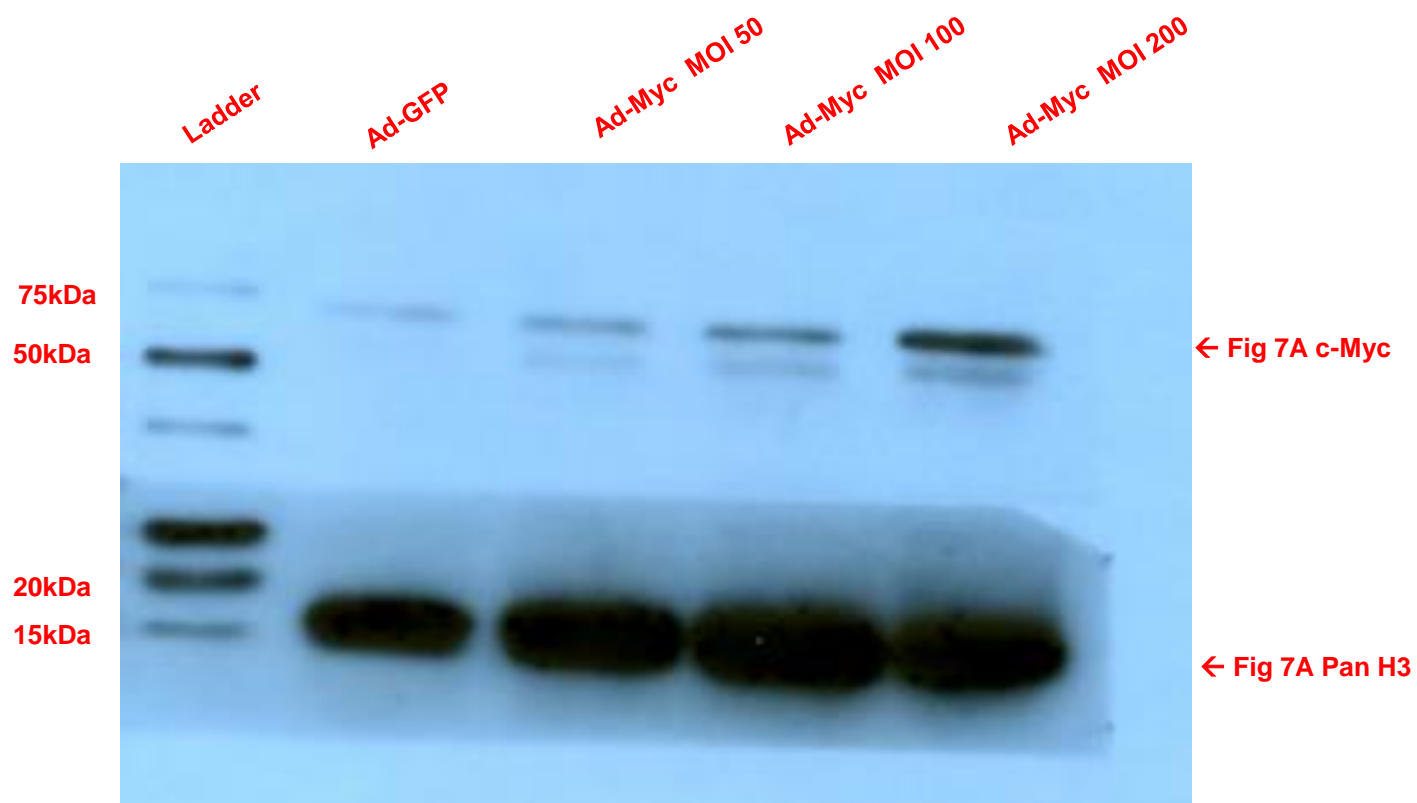

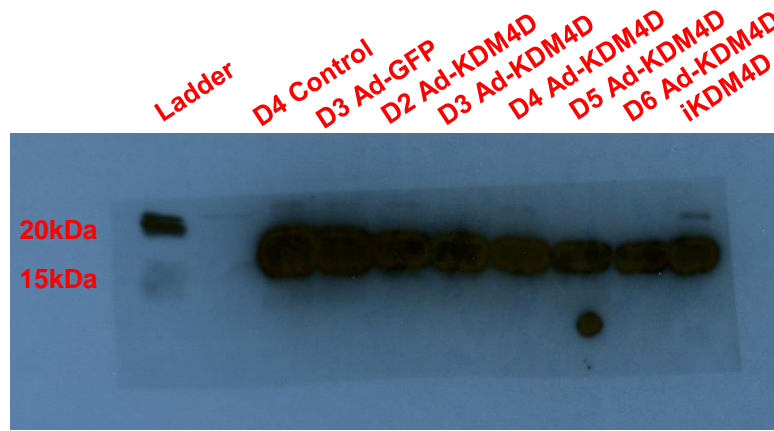

← S3 Fig PanH3

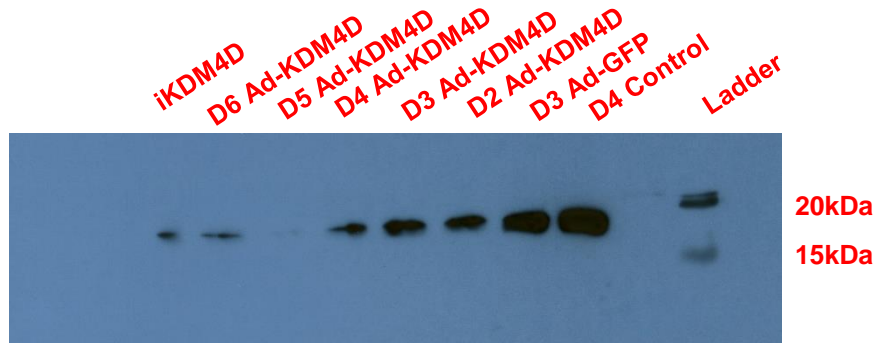

S3 Fig H3K9me3→

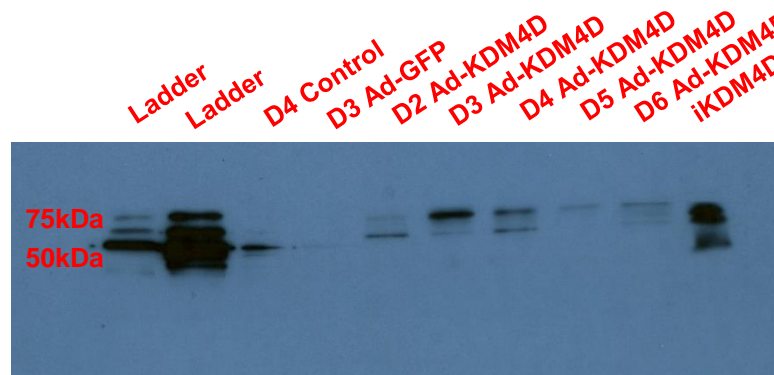

← S3 Fig KDM4D

Supplement: S1 Raw images — (PDF) [file pone.0281610.s010.pdf]
